# A Telemedicine System Using the Remote Diagnostic Imaging App “Join” for Emergency Medicine Throughout Wakayama Prefecture in Japan

**DOI:** 10.7759/cureus.69080

**Published:** 2024-09-10

**Authors:** Koji Fujita, Hiroyuki Takao, Seiya Kato, Naoyuki Nakao, Masami Ueno, Takako Nojiri

**Affiliations:** 1 Neurological Surgery, Naga Municipal Hospital, Kinokawa, JPN; 2 Innovation for Medical Information Technology, Jikei University School of Medicine, Tokyo, JPN; 3 Critical Care Medicine, Arida Municipal Hospital, Arida, JPN; 4 Neurological Surgery, Wakayama Medical University, Wakayama, JPN; 5 Community Medical Support Center, Wakayama Medical University, Wakayama, JPN; 6 Health and Social Welfare, Wakayama Prefectural Government, Wakayama, JPN

**Keywords:** emergency medicine, information and communications technology, regional medicine, remote image diagnosis system, smartphone application, telemedicine

## Abstract

Background

Telemedicine is expected to play an increasingly important role in building universal emergency medical systems without geographical disparities in the future. To eliminate healthcare disparities between regions, Wakayama Prefecture in Japan has been promoting telemedicine for closer inter-hospital coordination with secondary and tertiary emergency medical care hospitals. The “Join” diagnostic image-sharing app has been used since November 2018 for operating a remote emergency support system that shares medical images of emergency patients between 13 secondary and tertiary emergency medical care hospitals in Wakayama Prefecture. In this study, we investigated the effects of remote emergency medicine between hospitals after the introduction of Join.

Methodology

Four medical specialties use the remote emergency support system, namely, cardiovascular medicine, cardiovascular surgery, neurosurgery, and emergency care medicine. We investigated the results from 497 cases for which the system was used between November 2018 and February 2023.

Results

Of the 497 cases, treatment for 148 (29.8%) patients was continued in the same hospital, without a transfer. Emergency medicine was the group with the largest number of uses, in 232 cases. Of the 211 patients in the neurosurgery group, 88 (41.7%) were not transferred, the statistically significantly largest number of patients among the usage group (p < 0.01). An estimated transportation cost of 6,973,900 yen was saved due to 148 patients not being transferred. Tertiary emergency medical care hospitals were most frequently consulted, but secondary emergency medical care hospitals located long distances from tertiary emergency medical care hospitals also received large numbers of consultations from neighboring rural hospitals.

Conclusions

The Wakayama Prefecture remote emergency support system using Join has made it possible to communicate accurate information quickly between hospitals. This has contributed to fewer non-essential transfers to tertiary medical centers, reduced medical transport costs, and more effective distribution of medical resources to secondary medical institutions because ambulance transport was not necessary.

## Introduction

In Japan, there is a strong tendency for doctors to be concentrated in urban areas. This has caused a healthcare disparity in which residents of mountainous or remote areas cannot receive adequate healthcare as local services continue to collapse [1]. Telemedicine is being promoted as one means to resolve this problem. Telemedicine uses the Internet and other communication networks, as well as information and communication technology (ICT) such as information terminals, to provide medical examinations, diagnoses, and health consultations between geographically separated regions [2].

Telemedicine has been actively introduced in the field of emergency medicine. A wide variety of trials has been conducted, including 12-lead electrocardiograms that are sent from ambulances and used in the diagnosis and early treatment of myocardial infarction during transport [3,4], and smartphone positioning systems for patients having out-of-hospital cardiac arrest so that nearby citizen volunteers can be sent to the site to improve the cardiopulmonary resuscitation implementation rate [5].

Join is an image-sharing app for healthcare workers that has received the first medical certification in Japan. Medical images are displayed on smartphones or tablets and used in consultations between doctors. This enables coordination inside and outside the hospital and remote diagnosis and guidance. Outside of Japan, medical device certification has been obtained in the United States (FDA), Europe (CE), Brazil, Saudi Arabia, and elsewhere. In the treatment of acute stroke, the use of Join is reported to contribute to the early start of treatment, shorter hospital stays, improved outcomes, and lower medical expenses [6-14].

Wakayama Prefecture is located in the southwest part of the Kinki region of Japan. It is about 94 km east to west and about 106 km north to south, with an area of approximately 4,700 km^2^. More than 80% of the prefecture is mountainous. The population is 960,000, with 430,000 concentrated in the Wakayama medical area (340 km^2^) in the northeastern part of the prefecture. The only arterial highways are along the Kinokawa River that runs east to west in the northern part of the prefecture along the coast. Therefore, ambulance transport of emergency patients in the mountain regions can take a long time. In addition, about 50% of medical institutions and about 60% of doctors are concentrated in the Wakayama medical area at the northwest edge. Wakayama Prefecture has a total of three tertiary emergency medical care hospitals, namely, Wakayama Medical University Hospital (WMUH), Japanese Red Cross Wakayama Medical Center (RCWC), and Minami Wakayama Medical Center. WMUH and RCWC are in the Wakayama medical area.

Since November 2018, Wakayama Prefecture has operated a remote emergency support system that uses Join to share medical images of emergency patients with 13 secondary and tertiary emergency medical care hospitals, centered on WMUH. The purpose is to promote telemedicine, centered on strengthening inter-hospital coordination between secondary and tertiary emergency medical care hospitals across the entire prefecture. This was the first attempt in Japan to build such a system for an entire prefecture. As evidence of the effectiveness of regional coordination using Join, there is a report stating that it contributed to the reduction of non-essential transport of patients with neurosurgical emergencies to a tertiary emergency medical care hospital [13], but there are no reported analyses on the effectiveness of Join in networks covering all emergency medicine in a region. In this study, we examined the effectiveness of remote emergency medical care between hospitals after this system that utilizes real-time ICT was built.

## Materials and methods

Join usage policy: system construction

**Figure 1:**
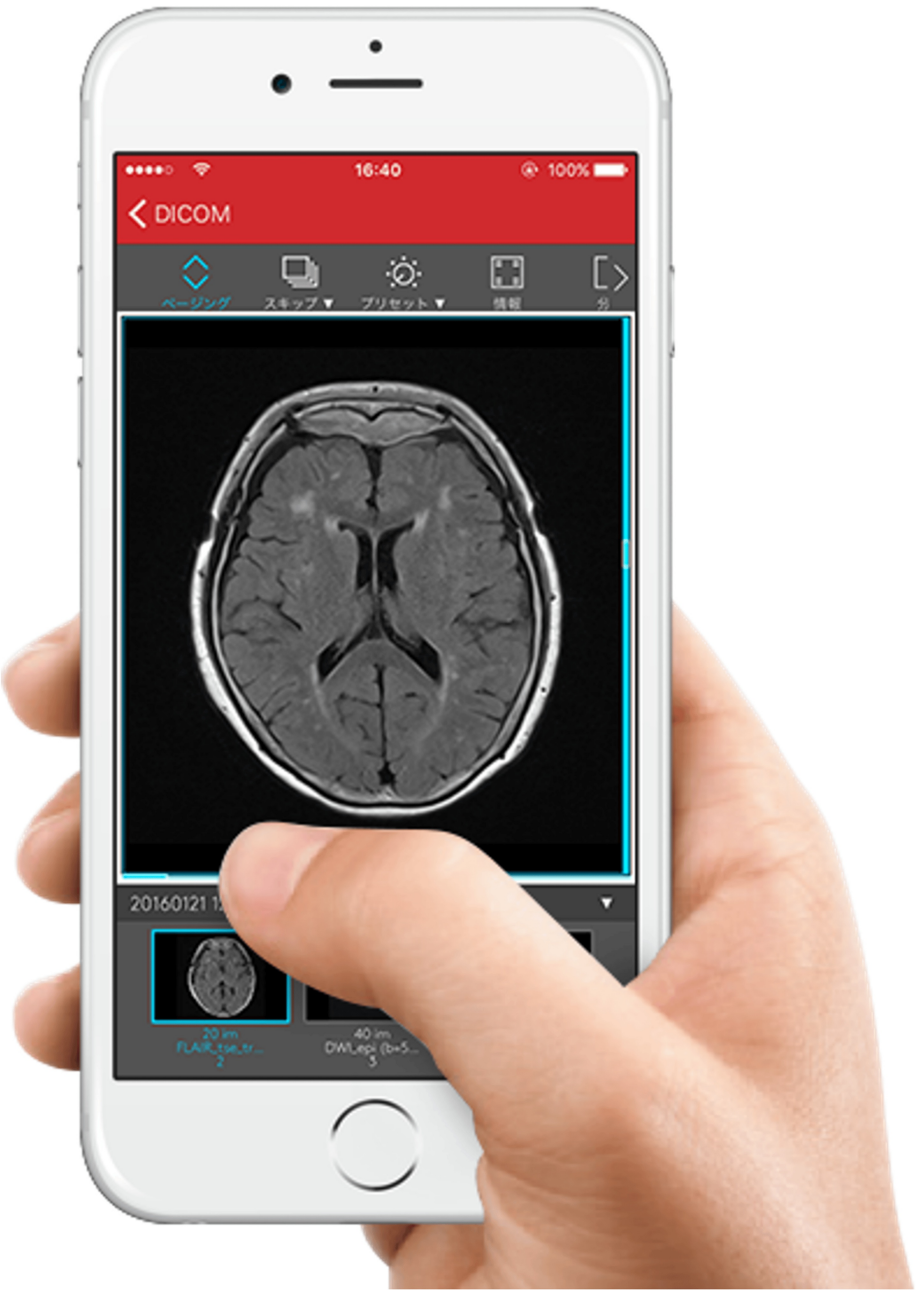
The remote image diagnosis support system “Join.” Users of Join can share information and images in a Digital Imaging and Communications in Medicine format on the high-resolution screens of smartphones and tablets. This sample image was provided with permission by Allm, Inc., Tokyo, Japan.

**Figure 2:**
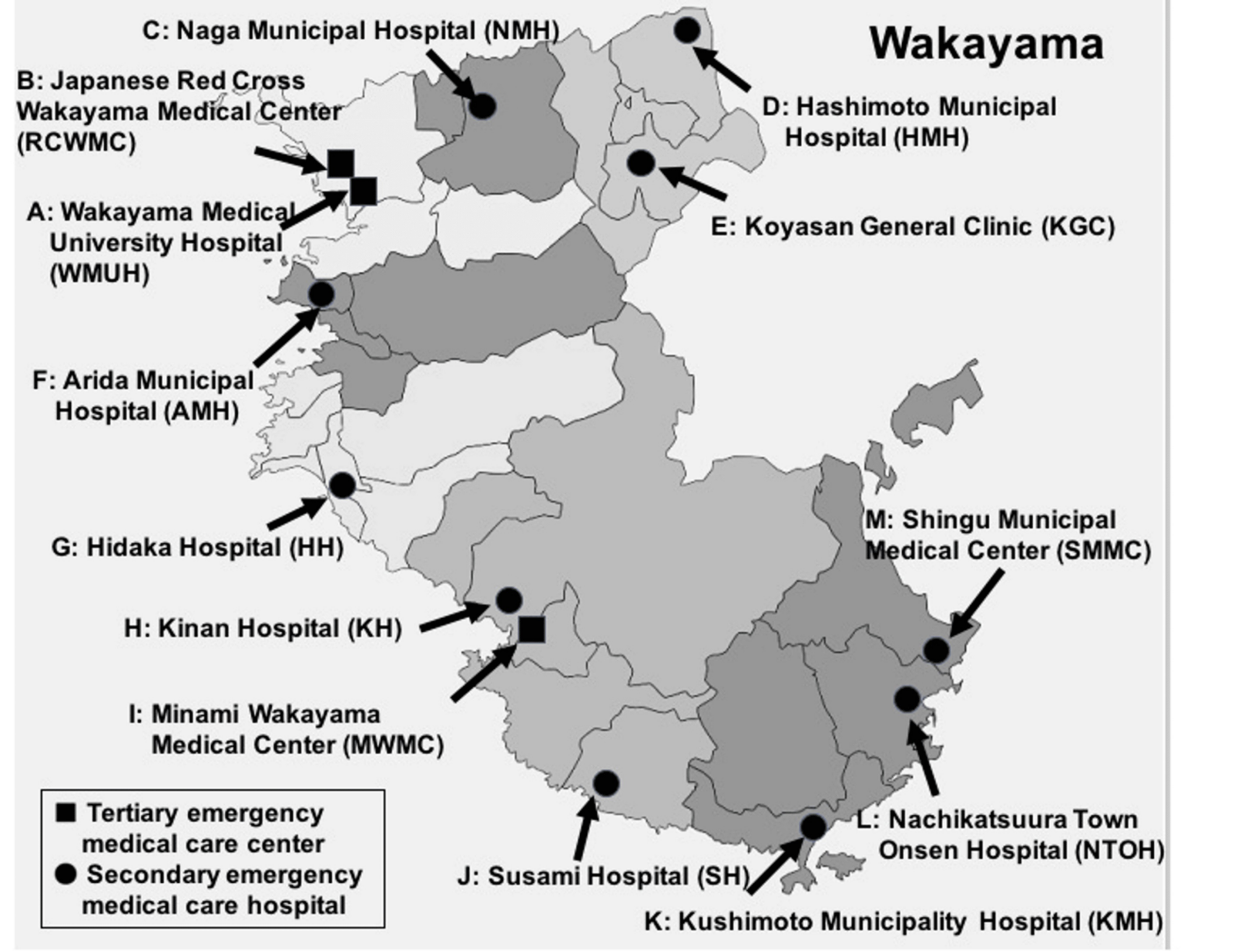
Geographical relationship of all hospitals in the prefecture-wide network participating in the Join remote image diagnosis support system.

The Wakayama Association for the Promotion of Telemedicine was established in May 2017 to promote telemedicine in Wakayama Prefecture. In this Association, 13 secondary and tertiary emergency medical care hospitals centered on WMUH agreed and obtained approval to build a remote emergency support system using Join (Figures 1, 2). The various rules shown below were established, and the system was put into operation in December 2018.

Before using Join, telephone contact was always made with the hospital receiving a consultation request, and confirmation was made that communication between hospitals was possible at the current time. When uploading information to the Join cloud, patient name, date of birth, and patient ID with Digital Imaging and Communications in Medicine tags are deleted to protect personal information. The data input for patient identification are (1) date and time (Western calendar), (2) name of medical institution, (3) age (decade of life), and (4) sex. The medical institutions participating in this system properly manage the medical records of all patients that are stored in Join and ensure that they are not leaked outside the system. Specifically, image data for a given patient that are on the Join cloud server are deleted at midnight after more than 48 hours have passed since they were uploaded to the server.

**Figure 3:**
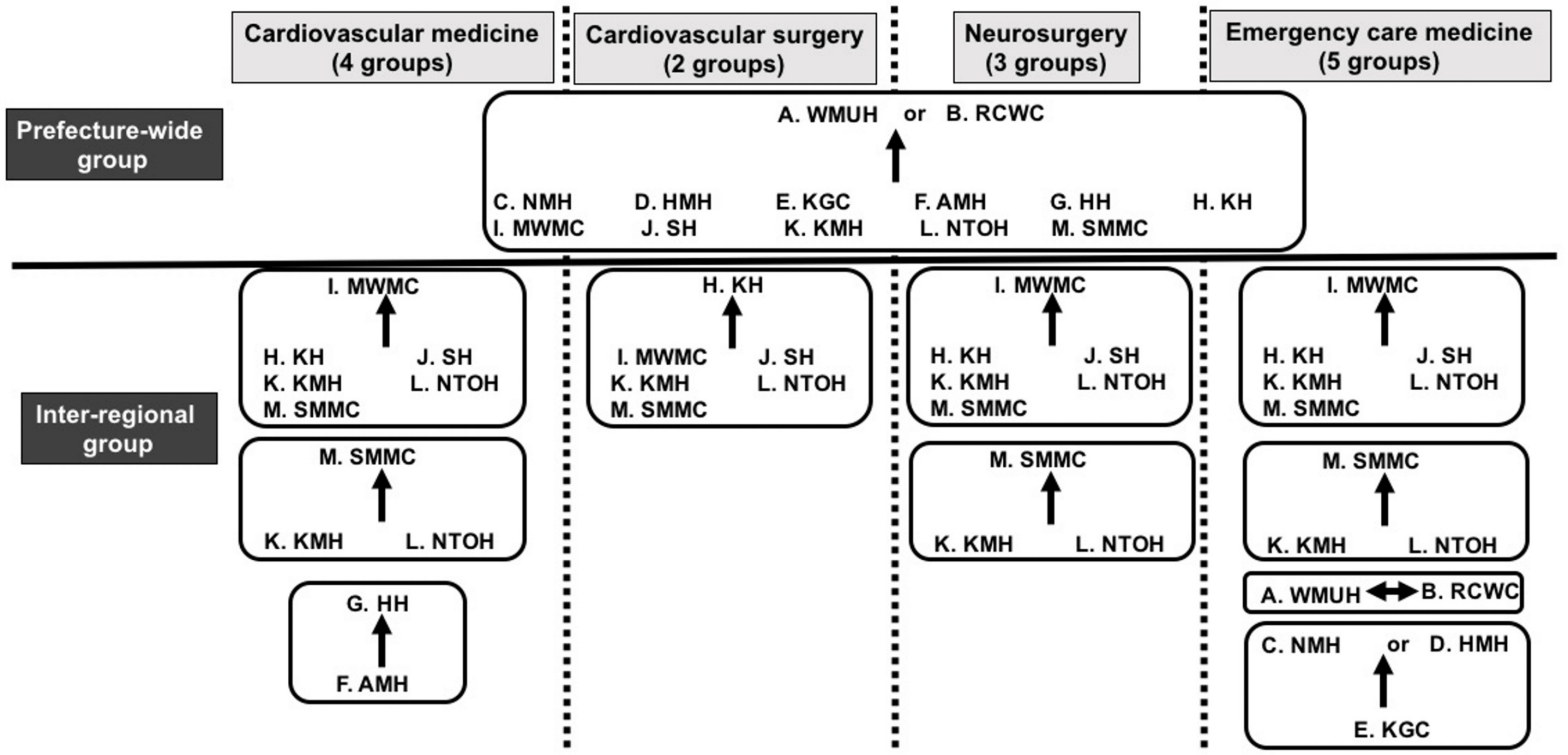
Telemedicine group using “Join” in the prefecture-wide network. Prefecture-wide group: The tertiary emergency medical care center, WMUH or RCMC, receives patient consultations from another 11 hospitals. Inter-regional group: In small groups established in line with regional characteristics for each department, core hospitals receive consultations from some rural hospitals in the area. Hospital abbreviations are the same as in Figure 2.

It was decided that the diseases covered by the remote emergency support system would be (1) stroke, (2) acute coronary syndrome, (3) acute aortic dissection, and (4) diseases for which there is an agreement for support between hospitals (e.g., severe head trauma, acute abdomen). The four specialties using the remote emergency support system were cardiovascular medicine, cardiovascular surgery, neurosurgery, and emergency care medicine. As the basic structure, a “prefecture-wide group” was built in which the tertiary emergency medical care hospitals WMUH and RCWC receive consultations from other hospitals (Figure 3).

Secondary emergency medical care hospitals to which emergency patients are transported by ambulance send computed tomography or magnetic resonance images using Join. The tertiary emergency medical care hospital that receives those images consults on the case while looking at the diagnostic images and decides whether the patient should be transferred. If it is determined that the case can be handled at the hospital sending the images, treatment guidance and advice are given. In cases when a transfer is accepted, arrangements for emergency procedures, emergency surgery, or other measures are rapidly made so that treatment can be started soon after the patient arrives. For patients who are not severe enough for consultation with a tertiary emergency medical care hospital, an inter-regional group is used. The number of specialists in each medical department and the emergency care system differs depending on the hospital, and so each of the four medical specialties is divided into small groups (Figure 3).

Items investigated

The number of times Join was used, the time of day it was used, the day it was used, whether the patient was transferred after the image consultation, the number of image consultations sent and received by each hospital, and the usage results in each group of each medical specialty were investigated from the time the system was started on November 1, 2018, until February 28, 2023. The time of day of use was classified as daytime (9:00 am to 5:00 pm) and nighttime (5:00 pm until 9:00 am the following day), and the day of use was classified as weekday, weekday before a holiday, or holiday. Fisher’s exact probability test was used for the statistical analysis and a p-value <0.05 was taken to indicate a statistically significant difference. EZR software was used for the statistical analysis [15].

For non-transferred patients, the round-trip ambulance transport distance, transport time, and ambulance transport cost if the patient had been transported by ambulance to the consulted hospital were calculated. The distance between the hospitals, round-trip time, and cost are shown in Table 1. The transport time was calculated assuming an ambulance speed of 50 km/hour and a time of 15 minutes spent at the destination hospital. The ambulance transport cost was calculated from the round-trip distance between hospitals with an ambulance fuel consumption of 4 km/L and gasoline price of 168 yen/L, in addition to the cost of 45,000 yen to dispatch the ambulance.

**Table 1:** One-way distance and round-trip time and cost required for ambulance transport between hospitals. Hospital abbreviations correspond to those in Figure 2.

	A. WMUH	B. RCWC	C. NMH	D. HMH	E. KGC	F. AMH	G. HH	H. KH	I. MWMC	J. SH	K. KMH	L. NTOH	M. SMMC	
A. WMUH	N/A	4.7	31.9	55.4	61.5	20.6	41.3	69.1	74.6	96.6	131	158	164	One-way distance (km)
B. RCWC	0.438/45,394.8	N/A	21.6	51.6	57.7	32.6	50.6	78.7	84.2	106	140	167	174	
C. NMH	1.526/47,679.6	1.114/46,814.4	N/A	32.7	33.4	47.8	65.9	93.9	99.4	121	155	182	189	
D. HMH	2.466/49,653.6	2.314/49,334.4	1.558/47,746.8	N/A	30.3	72.2	90.2	118	124	146	180	207	214	
E. KGC	2.71/50,166.6	2.558/49,846.8	1.586/47,805.6	1.462/47,545.2	N/A	77.9	95.9	124	129	151	185	161	152	
F. AMH	1.074/46,730.4	1.554/47,738.4	2.162/49,015.2	3.138/51,484.8	3.366/51,543.6	N/A	31.5	59.6	65.1	87.1	121	149	155	
G. HH	1.902/48,469.2	2.274/49,250.4	2.886/50,535.6	3.858/52,576.8	4.086/53,055.6	1.51/47,646	N/A	34	39.4	61.4	95.3	123	129	
H. KH	3.014/50,804.4	3.398/51,610.8	4.006/52,887.6	4.97/54,912	5.21/55,416	2.634/50,006.4	1.61/47,856	N/A	4.8	28.6	62.7	90.4	88.2	
I. MWMC	3.234/51,266.4	3.618/52,072.8	4.226/53,349.6	5.21/55,416	5.41/55,836	2.854/50,468.4	1.826/48,309.6	0.442/45,403.2	N/A	26.5	60.3	88.4	94.2	
J. SH	4.114/53,114.4	4.49/53,904	5.09/53,904	6.09/57,264	6.29/57,684	3.734/52,316.4	2.706/50,157.6	1.394/47,402.4	1.31/47,226	N/A	39.2	67.3	73	
K. KMH	5.49/56,004	5.85/56,760	6.45/58,020	7.45/60,120	7.65/60,540	5.09/55,164	4.062/53,005.2	2.758/50,266.8	2.662/50,065.2	1.818/48,292.8	N/A	29.4	36.2	
L. NTOH	6.57/58,272	6.93/59,028	7.53/60,288	8.53/62,388	6.69/58,524	6.21/57,516	5.17/55,332	3.866/52,593.6	3.786/52,425.6	2.942/50,653.2	1.426/47,469.6	N/A	10.6	
M. SMMC	6.81/58,776	7.21/59,616	7.81/60,876	8.81/62,976	6.33/57,768	6.45/58,020	5.41/55,836	3.778/52,408.8	4.018/52,912.8	3.17/51,132	1.698/48,040.8	0.674/45,890.4	N/A	
	Round-trip time (h) / cost (yen)													

## Results

The remote emergency support system was used in a total of 697 cases between November 1, 2018, and February 28, 2023. Excluding 72 cases for which it was used for consultation within a hospital and 128 cases for which there was insufficient information to conduct data analysis, 497 cases were analyzed.

Patient characteristics

Looking at whether patients were transported by ambulance after consultation, we see that the patient was not transferred in 148 (29.8%) of the 497 cases in which this system was used, but instead, continued to be treated at the same hospital (Table 2). By age, a statistically significantly larger number of younger patients were transferred. As age increased, transfers became less common. Among the patients who were transferred, 38.7% were women, and among the patients who were not transferred, 50% were women, indicating that statistically significantly more women were not transferred. About 30% of consultations occurred during the nighttime hours. Regarding the day of use, consultations on a weekday before a holiday occurred at about the same rate as those on holidays. The most frequent usage group was emergency care medicine, whereas the least frequent was cardiovascular medicine. Concerning whether the patient was transferred, in the neurosurgery group, 88 (41.7%) of 211 patients were not transferred. Therefore, a statistically significantly larger number of patients were not transferred among the usage group.

**Table 2:** Baseline characteristics and comparison of transferred and non-transferred patients and estimated saving benefits by avoiding unnecessary ambulance transport. Statistical method: Fisher’s exact probability test. *: Variables showing significant differences.

	Total (n = 497)	Transferred (n = 349)	Non-transferred (n = 148)	P-value
Age (years) distribution				0.0187*
Less than 70, n	163	126	37	
70–79, n	134	95	39	
80–89, n	156	104	52	
Over 90, n	44	24	20	
Sex				0.0223*
Female, n (%)	209 (42.1)	135 (38.7)	74 (50.0)	
Consultation time				0.595
Daytime, n (%)	347 (69.8)	241 (69.1)	106 (71.6)	
Consultation day				0.874
Weekday, n	292	207	85	
Day before a holiday, n	101	69	32	
Holiday, n	104	73	31	
Consulted department				0.00000952*
Cardiovascular medicine, n	5	4	1	
Cardiovascular surgery, n	49	40	9	
Neurosurgery, n	211	123	88	
Emergency care medicine, n	232	182	50	
Saving benefits by avoiding ambulance transport				N/A
Round-trip distance by ambulance (km)	7,574.40	-	7,574.40	
Round-trip time by ambulance (hours)	188.5	-	188.5	
Cost of ambulance use (yen)	6,973,900	-	6,973,900	

Differences by gender are shown in Table 3 and Table 4. For the age distribution, there was a significantly larger number of older patients among women (Table 3). Although no gender differences were seen in any of the usage groups, on the question of whether the patient was transferred, a significantly larger number of women (53.2%) in the neurosurgery group were not transferred (Table 4).

**Table 3:** Gender differences in age distribution. Statistical method: Fisher’s exact probability test. *: Variables showing significant differences.

Age (years) distribution	Total (n = 497)	Male (n = 288)	Female (n = 209)	P-value
Less than 70, n	163	109	54	0.0.00002196*
70–79, n	134	87	47	
80–89, n	156	78	78	
Over 90, n	44	14	30	

**Table 4:** Gender differences between transferred and non-transferred patients in consulted departments. Statistical method: Fisher’s exact probability test. *: Variables showing significant differences.

Consulted department	Total (n = 497)	Male (n = 288)		P-value	Female (n = 209)		P-value
		Transferred (n = 214)	Non-transferred (n = 74)		Transferred (n = 135)	Non-transferred (n = 74)	
Cardiovascular medicine, n	5	3	0	0.1576	1	1	0.00000492*
Cardiovascular surgery, n	49	21	6		19	3	
Neurosurgery, n	211	79	38		44	50	
Emergency care medicine, n	232	111	30		71	20	

Cost savings from not transferring patients

If the 148 non-transferred patients had been transported by ambulance to the consulted hospital, it was calculated that the time needed for that transport would have been 188.5 hours, at a cost of 6,973,900 yen. This cost was effectively eliminated by not transferring these patients (Table 2).

Inter-hospital consultation

The number of consultations between hospitals and the number of patients transferred are shown in Table 5. The hospital that received the most consultations (n = 169) was WMUH. Among these, transfer was not needed for 38 (22.5%) patients. Tertiary emergency medical care hospitals, Minami Wakayama Medical Center and RCWC, also received large numbers of consultations. Of the secondary emergency medical care hospitals that received many consultations, Hashimoto Municipal Hospital on the northeast edge of Wakayama Prefecture received 77 consultations and Shingu Municipal Medical Center on the southeast edge received 138 consultations. All of those consultations were from hospitals in neighboring rural areas. Six hospitals (Koyasan General Clinic, Arida Municipal Hospital, Hidaka Hospital, Susami Hospital, Kushimoto Municipality Hospital, and Nachikatsuura Town Onsen Hospital) located relatively long distances from tertiary emergency medical care hospitals did not receive any consultations; rather, they only requested consultations.

**Table 5:** Number of cases using the “Join” remote image diagnosis support system between hospitals. Hospital abbreviations are consistent with Figure 2.

	A. WMUH	B. RCWC	C. NMH	D. HMH	E. KGC	F. AMH	G. HH	H. KH	I. MWMC	J. SH	K. KMH	L. NTOH	M. SMMC	Total	
A. WMUH	N/A	17 (12)	-	-	-	-	-	-	-	-	-	-	-	17 (12)	Number of consultation requests (transferred cases)
B. RCWC	11 (9)	N/A	-	-	-	-	-	-	-	-	-	-	-	11 (9)	
C. NMH	13 (10)	-	N/A	-	-	-	-	-	-	-	-	-	-	13 (10)	
D. HMH	13 (9)	-	-	N/A	-	-	-	-	-	-	-	-	-	13 (9)	
E. KGC	7 (6)	-	4 (3)	77 (69)	N/A	-	-	-	-	-	-	-	-	88 (78)	
F. AMH	11 (5)	1 (1)	-	-	-	N/A	-	-	-	-	-	-	-	12 (6)	
G. HH	76 (62)	4 (3)	-	-	-	-	N/A	-	-	-	-	-	-	80 (65)	
H. KH	9 (4)	1 (1)	-	-	-	-	-	N/A	43 (26)	-	-	-	-	53 (31)	
I. MWMC	15 (13)	-	-	-	-	-	-	2 (1)	N/A	-	-	-	-	17 (14)	
J. SH	2 (2)	-	-	-	-	-	-	1 (1)	3 (2)	N/A	-	-	-	6 (5)	
K. KMH	7 (7)	-	-	-	-	-	-	-	26 (25)	-	N/A	-	38 (27)	71 (59)	
L. NTOH	2 (1)	-	-	-	-	-	-	1 (1)	7 (3)	-	-	N/A	100 (41)	110 (46)	
M. SMMC	3 (3)	-	-	-	-	-	-	1 (1)	2 (1)	-	-	-	N/A	6 (5)	
Total	169 (131)	23 (17)	4 (3)	77 (69)	-	-	-	5 (4)	81 (57)	-	-	-	138 (68)	497 (349)	
	Number of consultations received (transferred cases)														

## Discussion

Join is the first communications app for medical professionals that was certified and covered by health insurance in Japan as a medical device software program. It has been available for use in healthcare insurance treatment since April 2016. Occasional reports are seen on its usefulness in the sharing of necessary information when a patient is transferred between hospitals, and in information sharing between doctors within a hospital [6-14].

The remote emergency support system of secondary and tertiary emergency medical care hospitals in all of Wakayama Prefecture reduced ambulance transport in this series by 29.8% (Table 2). Thus, it was shown that reducing non-essential ambulance transport to tertiary emergency medical care hospitals contributes to the effective distribution of medical resources at secondary and tertiary emergency medical care hospitals, and the alleviation of excessive work burdens on physicians at tertiary medical care hospitals. A previous study reported that the use of Join between regional secondary and tertiary emergency medical care hospitals for acute neurosurgical diseases was effective in reducing non-essential transfer to tertiary emergency medical care hospitals and for local coordination [13]; however, in the present study, we confirmed the effectiveness of Join comprehensively for overall emergency medical care in a region.

One result of this study was that a significantly larger number of women were not transferred. A significantly larger number of older women, and those in the neurosurgery group, in particular, were not transferred (Tables 3, 4). This is conjectured to be because, among women, there were many older patients with stroke, many of whom had mild conditions that could be treated at the hospital seeking consultation with appropriate advice from an advanced medical institution. By contrast, it is thought that many men were transferred because they were young and had severe trauma.

For the day of use, almost as many consultations were seen on the weekday before a holiday (101 patients, 20.3%) as on holidays (104 cases, 20.9%). This indicates that secondary emergency medical care hospitals sought advice from tertiary medical care centers on the day before a weekend concerning whether the secondary emergency medical care hospital would be able to manage severe patients on weekends.

The most frequent usage group was emergency care medicine, followed by neurosurgery. In the neurosurgery group, 41.7% of patients were not transferred. Nearly all of the patients who required transfer were those who needed craniotomy or a procedure such as mechanical thrombectomy for acute ischemic stroke. Most patients who were not transferred had mild conditions that did not require surgery, such as lacunar infarction, intracerebral hemorrhage with a small amount of hematoma, head trauma without impaired consciousness, or stroke mimics including epilepsy. Considering that before the introduction of Join, all of these patients were transferred, it indicates that in the neurosurgery group, appropriate diagnosis and advice from the hospital receiving the consultation with the introduction of Join reduced unnecessary transfers, and as a result, contributed greatly to the alleviation of excessive burdens on physicians at tertiary emergency medical care hospitals.

In an analysis of the number of consultations between hospitals, those receiving the most consultations were WMUH and other tertiary emergency medical care hospitals, but many consultations were also received by Hashimoto Municipal Hospital on the northeast edge of Wakayama Prefecture and Shingu Municipal Medical Center on the southeast edge, all of which were from neighboring rural hospitals. With the effective use of Join within inter-regional groups, good coordination was built between rural hospitals and secondary emergency medical care hospitals. In particular, Nachikatsuura Town Onsen Hospital consulted Shingu Municipal Medical Center on 100 patients, of whom only 41 were transferred. It was understood that Nachikatsuura Town Onsen Hospital depends strongly on Shingu Municipal Medical Center for diagnosis and treatment policy guidance, and Shingu Municipal Medical Center responds to those needs. By contrast, mutual consultations of more than 10 cases each also occurred between WMUH and RCWC, which are neighboring tertiary emergency medical care hospitals (Table 5). From this result, it is understood that although patients were transferred to a tertiary emergency medical care hospital, Join was used for consultation with another nearby tertiary emergency medical care hospital in situations when managing admission to the same hospital would be difficult for reasons such as unavailability of surgical staff and a lack of intensive care unit beds. In other words, it was used to manage beds between tertiary emergency medical care hospitals. Six hospitals (Koyasan General Clinic, Arida Municipal Hospital, Hidaka Hospital, Susami Hospital, Kushimoto Municipality Hospital, and Nachikatsuura Town Onsen Hospital) located relatively far distances from a tertiary emergency medical care hospital did not receive any consultations. Rather, they only requested consultations and seemed to depend strongly on advanced medical institutions. With the advice these hospitals received from advanced medical institutions, many patients could continue to receive care at the same hospital without being transferred. In this way, it was demonstrated that medical resources were effectively distributed at secondary and tertiary medical care hospitals with the introduction of Join.

Here, we look at the effects produced, in terms of cost, from the 148 cases in this series in which the patient was not transferred. Considering that before the introduction of Join, all of these patients would have been transferred to an advanced medical institution, we calculate that the cost would have been 6,973,900 yen from a total of 188.5 hours of ambulance transportation and the mobilization of local paramedics for the transport. This reduction contributes greatly to alleviating the burden on paramedics. Transport by sending an ambulance to a remote area means that there is one less ambulance within the local healthcare district for that time. If multiple ambulance requests are made during this period, there is a possibility that responding to them would be difficult. Therefore, limiting long-distance transport by ambulance between hospitals after the introduction of Join has also served a role in the effective use of ambulances. It has also alleviated the burden of being transported to a distant hospital on the patients themselves, as well as the burden on the families of the patients being admitted to a hospital far from their homes.

A limitation of this study is that it was conducted in the specific region of Wakayama Prefecture, so the results may not be applicable to all other regions. In emergency medicine, both the population and medical resources, including the distribution of hospitals to which patients are transported, differ depending on the region. In Wakayama Prefecture, despite the small number of emergency medical facilities and specialists in the prefecture as a whole, the area to be covered is large and the transportation network for ambulance transport is poorly developed. Geographically, the provision of universal medical care is difficult over the entire prefecture. Even so, the collapse of community medical care, in which emergency medicine that has become standard in urban areas is not provided to these residents as they live in isolated areas, must be avoided. The local government in Wakayama Prefecture is promoting telemedicine from this perspective and has succeeded in putting this system into operation.

The second limitation is that we did not investigate whether patient outcomes were improved with the introduction of this system. This study mainly examined the effect of the reduction in non-essential ambulance transport after the introduction of Join. However, appropriate treatment could be started quickly at the local hospital, even for patients who were not transferred, based on the advice received from an advanced medical institution. For those patients who are transferred to an advanced medical institution, it has become possible to conduct emergency procedures, emergency surgery, or other preparations so that treatment can begin soon after the patient arrives. In fact, in neurological emergencies, the usefulness of Join has been felt in consultations with regional hospitals that cannot perform mechanical thrombectomy for acute ischemic stroke regarding whether mechanical thrombectomy should be performed. We have experienced several cases in which mechanical thrombectomy is judged to be indicated, intravenous recombinant tissue plasminogen activator is started at the consulting hospital, the hospital being consulted makes preparations for mechanical thrombectomy during drip and ship transport before the patient arrives, and a good outcome is achieved with mechanical thrombectomy soon after the patient arrives. Therefore, the introduction of this system is thought to have a huge significance. The use of Join by staff within a hospital contributes to the early start of treatment for acute revascularization [9,11,14]. It has also been reported that using methods such as image sharing or voice and video calls and having multiple staff members from remote locations view intraoperative images helps to ensure treatment quality [16].

The third limitation is that ambulance transport was assumed to calculate the costs saved by not transporting patients. Wakayama Prefecture introduced emergency medical helicopters in 2003, which has contributed to emergency medicine centered on WMUH for patients in mountainous and remote areas [17,18]. After the introduction of Join, proper judgments on whether to use ambulance or helicopter transport have been made by sharing patient information and images between hospitals. As image information could not be shared before the start of Join, there were cases of patients being transported by emergency medical helicopters even when transport was unnecessary [19]. Particularly when consultations are made from a hospital a long distance from a tertiary emergency medical care hospital, there is a tendency to opt for emergency medical helicopter transport. The choice of ambulance or helicopter transport is made with discussions between doctors at different hospitals. Of the 148 patients in this series who were not transferred, it is difficult to assess how many would have been transported by emergency medical helicopter from only the data collected here. Therefore, in this study, we attempted to calculate the reduction in costs assuming all transportation by ambulance.

The final problem is a plan for the continuation of this system. The current system has few advantages for the hospitals accepting the patients. Advanced medical institutions that receive the sent images have few incentives and accept consultations free of charge. From the perspective of protecting personal information, it may also be considered a problem that the exchanges between hospitals on Join are not kept as medical records. Specifically, the advanced medical institutions being consulted do not produce a patient ID, and so do not generate any medical records.

That being said, further progress in using ICT should be made from the perspectives of the quality of medical care and the reduction in medical costs. Quickly sharing medical images over long distances holds potential for the major expansion of emergency medicine. This is backed by the spread of smartphones and the growth of communication networks. No effort should be spared to promote emergency medicine with the use of ICT, including the establishment of standards for the handling of medical information and national support for things such as running costs.

## Conclusions

Using the Join remote diagnostic imaging app, a remote emergency support system that shares medical images of emergency patients with 13 secondary and tertiary emergency medical care hospitals in all of Wakayama Prefecture has been built. With the use of this system, about 30% of patients are not transferred to advanced medical institutions but continue to receive treatment at the original hospital. Join has made it possible to communicate accurate information quickly between hospitals and contributed to a reduction in non-essential transfers to tertiary medical institutions, decreased costs for transport, and the effective distribution of medical resources to secondary medical care hospitals by not requiring patients to be transferred. Building a system that has benefits for the institutions to which images are sent is an issue for the future.

## Appendices

**Table 6:** Remote emergency support system usage record.

	Date and time of use	Day of week	Holiday	Night time (17:00～9:00）	Age (decade of life)	Gender	Speciality	Image source	Image destination	Whether or not to transfer	One-way travel distance (km)	Round-trip distance (km)	One-way travel time (50 km/hour)	Round-trip travel time (50 km/hour)	Ambulance cost (Yen)	Ambulance cost ($) 1$ = 141¥	Gasoline cost 168 yen/L(4 km/L)	Gasoline cost$ (168 yen/L) (4 km/L)	
																			1	2019/1/2 13:55	Wed.	Holiday		80	Male	EmergentCare M.	AMH	WMUH	No	20.6	41.2	0.412	1.074	45000	319.1489362	1730.4	12.27234043	
2	2019/1/16 10:57	Wed.			80	Female	Cardiovasc.Surg.	AMH	RCWC	Transfer									
3	2019/1/19 21:34	Sat.	Holiday	◯	80	Female	Neurosurg.	KMH	MWMC	transfer									
4	2019/2/5 9:55	Tue.			60	Female	Neurosurg.	AMH	WMUH	Transfer									
5	2019/2/24 21:20	Sun.	Holiday	◯	70	Female	Cardiovasc.Surg.	HH	WMUH	Transfer									
6	2019/3/2 19:20	Sat.	Holiday	◯	80	Female	Neurosurg.	HH	WMUH	Transfer									
7	2019/3/21 15:38	Thu.			80	Female	Cardiovasc.Surg.	HH	WMUH	Transfer									
8	2019/5/7 15:19	Tue.			20	Male	EmergentCare M.	KGC	HMH	No	30.3	60.6	0.606	1.462	45000	319.1489362	2545.2	18.05106383	
9	2019/5/18 12:42	Sat.	Holiday		70	Female	Neurosurg.	HH	WMUH	Transfer									
10	2019/6/1 22:37	Sat.	Holiday	◯	80	Female	EmergentCare M.	AMH	WMUH	Transfer									
11	2019/6/1 22:50	Sat.	Holiday	◯	80	Male	Cardiovasc.Surg.	HH	WMUH	No	41.3	82.6	0.826	1.902	45000	319.1489362	3469.2	24.60425532	
12	2019/7/4 13:10	Thu.			40	Male	CardioVasc.M.	WMUH	RCWC	Transfer									
13	2019/8/14 10:30	Wed.			60	Female	EmergentCare M.	KGC	HMH	Transfer									
14	2019/8/22 14:23	Thu.			60	Female	EmergentCare M.	KGC	HMH	Transfer									
15	2019/8/23 11:08	Fri.	Day before a holiday		80	Male	EmergentCare M.	KH	WMUH	No	69.1	138.2	1.382	3.014	45000	319.1489362	5804.4	41.16595745	
16	2019/9/2 11:00	Mon.			60	Male	EmergentCare M.	KGC	HMH	Transfer									
17	2019/9/13 12:30	Fri.	Day before a holiday		70	Male	Neurosurg.	KH	WMUH	No	69.1	138.2	1.382	3.014	45000	319.1489362	5804.4	41.16595745	
18	2019/9/19 19:19	Thu.		◯	10	Male	Neurosurg.	KGC	HMH	Transfer									
19	2019/9/27 12:53	Fri.	Day before a holiday		90	Male	Neurosurg.	NTOH	SMMC	No	10.6	21.2	0.212	0.674	45000	319.1489362	890.4	6.314893617	
20	2019/10/7 14:05	Mon.			40	Male	EmergentCare M.	KGC	HMH	Transfer									
21	2019/10/11 17:38	Fri.	Day before a holiday	◯	80	Female	Neurosurg.	AMH	WMUH	No	20.6	41.2	0.412	1.074	45000	319.1489362	1730.4	12.27234043	
22	2019/10/21 15:02	Mon.	Day before a holiday		60	Male	EmergentCare M.	KGC	HMH	Transfer									
23	2019/11/20 18:30	Wed.		◯	70	Female	Cardiovasc.Surg.	SMMC	WMUH	Transfer									
24	2019/12/9 2:47	Mon.		◯	10	Female	Neurosurg.	KH	WMUH	Transfer									
25	2019/12/17 16:55	Tue.			60	Male	EmergentCare M.	KMH	WMUH	Transfer									
26	2019/12/18 21:55	Wed.		◯	10	Female	EmergentCare M.	HH	WMUH	Transfer									
27	2019/12/22 5:56	Sun.	Holiday	◯	80	Female	Neurosurg.	HH	WMUH	Transfer									
28	2019/12/23 15:20	Mon.			80	Male	Neurosurg.	SH	MWMC	No	26.5	53	0.53	1.31	45000	319.1489362	2226	15.78723404	
29	2019/12/25 21:19	Wed.		◯	90	Female	EmergentCare M.	WMUH	RCWC	Transfer									
30	2020/1/7 15:19	Tue.			90	Male	EmergentCare M.	HH	WMUH	Transfer									
31	2020/1/14 10:54	Tue.			20	Female	EmergentCare M.	KGC	HMH	Transfer									
32	2020/1/14 13:03	Tue.			80	Female	EmergentCare M.	HH	WMUH	Transfer									
33	2020/1/14 17:24	Tue.		◯	80	Female	Neurosurg.	NTOH	SMMC	No	10.6	21.2	0.212	0.674	45000	319.1489362	890.4	6.314893617	
34	2020/1/22 11:00	Wed.			60	Male	Neurosurg.	KGC	HMH	Transfer									
35	2020/1/22 11:50	Wed.			60	Male	EmergentCare M.	KMH	WMUH	Transfer									
36	2020/1/29 17:10	Wed.		◯	70	Female	EmergentCare M.	KGC	HMH	Transfer									
37	2020/1/30 0:30	Thu.		◯	10	Male	EmergentCare M.	HH	WMUH	Transfer									
38	2020/1/31 16:06	Fri.	Day before a holiday		70	Female	Cardiovasc.Surg.	SH	WMUH	Transfer									
39	2020/2/1 19:48	Sat.	Holiday	◯	70	Male	Cardiovasc.Surg.	HH	WMUH	Transfer									
40	2020/2/6 22:39	Thu.		◯	80	Female	Neurosurg.	KMH	MWMC	Transfer									
41	2020/2/12 13:56	Wed.			70	Male	EmergentCare M.	KMH	MWMC	Transfer									
42	2020/2/26 12:30	Wed.			50	Male	EmergentCare M.	HH	WMUH	Transfer									
43	2020/3/3 17:13	Tue.		◯	80	Female	EmergentCare M.	SH	WMUH	Transfer									
44	2020/3/8 13:58	Sun.	Holiday		70	Female	Cardiovasc.Surg.	HH	WMUH	Transfer									
45	2020/3/9 17:51	Mon.		◯	80	Male	EmergentCare M.	NMH	WMUH	Transfer									
46	2020/3/10 18:23	Tue.		◯	60	Male	Neurosurg.	HH	WMUH	Transfer									
47	2020/3/11 19:37	Wed.		◯	70	Female	Neurosurg.	NTOH	SMMC	No	10.6	21.2	0.212	0.674	45000	319.1489362	890.4	6.314893617	
48	2020/3/13 11:21	Fri.	Day before a holiday		80	Male	EmergentCare M.	HH	WMUH	Transfer									
49	2020/4/4 9:59	Sat.	Holiday		30	Male	EmergentCare M.	MWMC	WMUH	Transfer									
50	2020/4/14 16:54	Tue.			40	Male	EmergentCare M.	MWMC	WMUH	Transfer									
51	2020/4/15 12:56	Wed.			80	Female	Neurosurg.	KH	MWMC	No	4.8	9.6	0.096	0.442	45000	319.1489362	403.2	2.859574468	
52	2020/4/17 12:48	Fri.	Day before a holiday		80	Male	Neurosurg.	KMH	MWMC	Transfer									
53	2020/4/23 0:52	Thu.		◯	70	Female	Neurosurg.	AMH	WMUH	No	20.6	41.2	0.412	1.074	45000	319.1489362	1730.4	12.27234043	
54	2020/4/26 9:17	Sun.	Holiday		40	Male	EmergentCare M.	MWMC	WMUH	Transfer									
55	2020/4/30 15:22	Thu.			80	Female	Cardiovasc.Surg.	AMH	WMUH	Transfer									
56	2020/5/6 17:51	Wed.	Holiday	◯	70	Male	Neurosurg.	HH	WMUH	Transfer									
57	2020/5/19 10:01	Tue.			60	Male	Neurosurg.	KGC	WMUH	Transfer									
58	2020/5/20 14:15	Wed.			70	Male	Neurosurg.	WMUH	RCWC	Transfer									
59	2020/5/22 19:59	Fri.	Day before a holiday	◯	10	Male	EmergentCare M.	AMH	WMUH	Transfer									
60	2020/6/4 21:23	Thu.		◯	50	Female	Neurosurg.	HH	WMUH	Transfer									
61	2020/6/6 10:36	Sat.	Holiday		70	Male	EmergentCare M.	AMH	WMUH	No	20.6	41.2	0.412	1.074	45000	319.1489362	1730.4	12.27234043	
62	2020/6/10 13:03	Wed.			80	Female	Neurosurg.	HH	WMUH	No	41.3	82.6	0.826	1.902	45000	319.1489362	3469.2	24.60425532	
63	2020/6/12 11:02	Fri.	Day before a holiday		70	Male	Cardiovasc.Surg.	KH	WMUH	Transfer									
64	2020/6/19 12:18	Fri.	Day before a holiday		60	Female	Cardiovasc.Surg.	MWMC	WMUH	No	74.6	149.2	1.492	3.234	45000	319.1489362	6266.4	44.44255319	
65	2020/6/20 23:33	Sat.	Holiday	◯	80	Male	Cardiovasc.Surg.	KMH	WMUH	Transfer									
66	2020/6/22 23:46	Mon.		◯	90	Male	EmergentCare M.	HH	RCWC	No	50.6	101.2	1.012	2.274	45000	319.1489362	4250.4	30.14468085	
67	2020/6/24 12:12	Wed.			70	Female	EmergentCare M.	HH	WMUH	Transfer									
68	2020/6/25 8:42	Thu.		◯	70	Male	EmergentCare M.	KGC	HMH	Transfer									
69	2020/6/26 10:12	Fri.	Day before a holiday		80	Male	EmergentCare M.	KGC	HMH	Transfer									
70	2020/6/29 9:37	Mon.			80	Male	EmergentCare M.	KGC	HMH	Transfer									
71	2020/6/29 14:00	Mon.			40	Female	EmergentCare M.	NMH	WMUH	Transfer									
72	2020/6/30 14:24	Tue.			50	Female	EmergentCare M.	MWMC	KH	Transfer									
73	2020/7/6 15:17	Mon.			10	Female	Neurosurg.	HH	WMUH	No	41.3	82.6	0.826	1.902	45000	319.1489362	3469.2	24.60425532	
74	2020/7/8 10:24	Wed.			80	Male	Neurosurg.	HH	WMUH	Transfer									
75	2020/7/9 15:51	Thu.			80	Male	EmergentCare M.	NMH	WMUH	No	31.9	63.8	0.638	1.526	45000	319.1489362	2679.6	19.00425532	
76	2020/7/18 13:43	Sat.	Holiday		80	Female	EmergentCare M.	HMH	WMUH	Transfer									
77	2020/7/21 10:11	Tue.			50	Male	EmergentCare M.	HMH	WMUH	Transfer									
78	2020/7/23 17:41	Thu.	Holiday	◯	90	Female	EmergentCare M.	KGC	HMH	Transfer									
79	2020/7/25 8:45	Sat.	Holiday	◯	60	Male	Neurosurg.	HH	WMUH	No	41.3	82.6	0.826	1.902	45000	319.1489362	3469.2	24.60425532	
80	2020/7/26 9:04	Sun.	Holiday		60	Male	EmergentCare M.	MWMC	WMUH	Transfer									
81	2020/7/28 13:38	Tue.			70	Male	CardioVasc.M.	HH	WMUH	Transfer									
82	2020/7/29 15:10	Tue.			80	Female	EmergentCare M.	WMUH	RCWC	No	4.7	9.4	0.094	0.438	45000	319.1489362	394.8	2.8	
83	2020/8/1 12:42	Sat.	Holiday		70	Male	EmergentCare M.	MWMC	WMUH	Transfer									
84	2020/8/14 9:26	Fri.	Day before a holiday		70	Female	EmergentCare M.	HMH	WMUH	Transfer									
85	2020/8/14 11:00	Fri.	Day before a holiday		70	Female	EmergentCare M.	NMH	WMUH	No	31.9	63.8	0.638	1.526	45000	319.1489362	2679.6	19.00425532	
86	2020/8/19 14:20	Wed.			70	Male	Neurosurg.	HH	WMUH	Transfer									
87	2020/8/20 21:21	Thu.		◯	60	Female	Neurosurg.	KH	MWMC	No	4.8	9.6	0.096	0.442	45000	319.1489362	403.2	2.859574468	
88	2020/8/27 6:53	Thu.		◯	60	Male	EmergentCare M.	MWMC	KH	No	4.8	9.6	0.096	0.442	45000	319.1489362	403.2	2.859574468	
89	2020/8/27 18:58	Thu.		◯	10	Male	Neurosurg.	HH	WMUH	Transfer									
90	2020/8/29 9:17	Sat.	Holiday		50	Male	EmergentCare M.	MWMC	WMUH	Transfer									
91	2020/9/2 2:11	Wed.		◯	80	Female	EmergentCare M.	SH	MWMC	Transfer									
92	2020/9/4 16:49	Fri.	Day before a holiday		90	Male	Cardiovasc.Surg.	HMH	WMUH	Transfer									
93	2020/9/13 8:57	Sun.	Holiday	◯	40	Male	EmergentCare M.	KMH	WMUH	Transfer									
94	2020/9/12 20:38	Sat.	Holiday	◯	60	Male	Neurosurg.	KH	MWMC	Transfer									
95	2020/9/15 17:05	Tue.		◯	10	Male	Neurosurg.	KGC	HMH	Transfer									
96	2020/9/17 14:46	Thu.			60	Male	EmergentCare M.	KGC	HMH	No	30.3	60.6	0.606	1.462	45000	319.1489362	2545.2	18.05106383	
97	2020/9/17 16:34	Thu.			70	Female	EmergentCare M.	KGC	HMH	Transfer									
98	2020/9/18 13:35	Fri.	Day before a holiday		80	Female	Neurosurg.	WMUH	RCWC	Transfer									
99	2020/9/23 12:08	Wed.			70	Male	EmergentCare M.	KGC	HMH	Transfer									
100	2020/9/24 19:24	Thu.		◯	60	Male	EmergentCare M.	KGC	HMH	Transfer									
101	2020/10/10 10:30	Sat.	Holiday		70	Male	EmergentCare M.	KGC	HMH	Transfer									
102	2020/10/19 19:37	Mon.		◯	60	Male	EmergentCare M.	MWMC	WMUH	Transfer									
103	2020/10/21 17:21	Wed.		◯	80	Female	EmergentCare M.	SH	MWMC	Transfer									
104	2020/10/30 14:08	Fri.	Day before a holiday		80	Female	Neurosurg.	KMH	MWMC	Transfer									
105	2020/11/2 18:24	Mon.	Day before a holiday	◯	60	Male	EmergentCare M.	HH	WMUH	Transfer									
106	2020/11/4 18:42	Wed.		◯	60	Male	Neurosurg.	HMH	WMUH	No	55.4	110.8	1.108	2.466	45000	319.1489362	4653.6	33.00425532	
107	2020/11/5 13:01	Thu.			10	Male	EmergentCare M.	HMH	WMUH	No	55.4	110.8	1.108	2.466	45000	319.1489362	4653.6	33.00425532	
108	2020/11/9 8:56	Mon.		◯	60	Male	Cardiovasc.Surg.	HH	WMUH	Transfer									
109	2020/11/10 16:56	Tue.			70	Male	EmergentCare M.	MWMC	WMUH	Transfer									
110	2020/11/12 10:51	Thu.			80	Female	Cardiovasc.Surg.	HMH	WMUH	No	55.4	110.8	1.108	2.466	45000	319.1489362	4653.6	33.00425532	
111	2020/11/13 9:28	Fri.	Day before a holiday		60	Male	Neurosurg.	KMH	MWMC	Transfer									
112	2020/11/18 10:16	Wed.			80	Female	EmergentCare M.	KMH	SMMC	No	36.2	72.4	0.724	1.698	45000	319.1489362	3040.8	21.56595745	
113	2020/11/18 11:13	Wed.			80	Male	Cardiovasc.Surg.	HH	WMUH	Transfer									
114	2020/11/19 16:57	Thu.			80	Male	Neurosurg.	WMUH	RCWC	Transfer									
115	2020/11/20 1:59	Fri.	Day before a holiday	◯	40	Male	EmergentCare M.	RCWC	WMUH	No	4.7	9.4	0.094	0.438	45000	319.1489362	394.8	2.8	
116	2020/11/20 16:17	Fri.	Day before a holiday		60	Male	EmergentCare M.	HH	WMUH	Transfer									
117	2020/11/24 10:44	Tue.			80	Female	Neurosurg.	KMH	SMMC	Transfer									
118	2020/12/2 12:01	Wed.			70	Female	Neurosurg.	MWMC	WMUH	Transfer									
119	2020/12/3 14:21	Thu.			80	Male	EmergentCare M.	KMH	SMMC	No	36.2	72.4	0.724	1.698	45000	319.1489362	3040.8	21.56595745	
120	2020/12/7 18:50	Mon.		◯	90	Female	EmergentCare M.	AMH	WMUH	No	20.6	41.2	0.412	1.074	45000	319.1489362	1730.4	12.27234043	
121	2020/12/10 15:44	Thu.			50	Male	Neurosurg.	HH	WMUH	No	41.3	82.6	0.826	1.902	45000	319.1489362	3469.2	24.60425532	
122	2020/12/11 8:41	Fri.	Day before a holiday	◯	80	Male	Cardiovasc.Surg.	HMH	WMUH	Transfer									
123	2020/12/13 18:58	Sun.	Holiday	◯	90	Female	Neurosurg.	NTOH	SMMC	No	10.6	21.2	0.212	0.674	45000	319.1489362	890.4	6.314893617	
124	2020/12/14 16:20	Mon.			80	Male	EmergentCare M.	HMH	WMUH	Transfer									
125	2020/12/15 17:14	Tue.		◯	70	Female	Cardiovasc.Surg.	HH	WMUH	Transfer									
126	2020/12/16 0:02	Tue.		◯	60	Female	EmergentCare M.	HH	WMUH	Transfer									
127	2020/12/17 15:55	Thu.			50	Female	Neurosurg.	HH	WMUH	No	41.3	82.6	0.826	1.902	45000	319.1489362	3469.2	24.60425532	
128	2020/12/22 11:22	Tue.			50	Female	Neurosurg.	NTOH	SMMC	Transfer									
129	2020/12/24 14:15	Thu.			50	Female	EmergentCare M.	HMH	WMUH	Transfer									
130	2020/12/31 16:02	Thu.	Holiday		70	Male	Neurosurg.	AMH	WMUH	No	20.6	41.2	0.412	1.074	45000	319.1489362	1730.4	12.27234043	
131	2021/1/1 12:11	Fri.	Holiday		80	Male	EmergentCare M.	KGC	HMH	Transfer									
132	2021/1/3 22:55	Sun.	Holiday	◯	90	Female	Neurosurg.	KH	MWMC	No	4.8	9.6	0.096	0.442	45000	319.1489362	403.2	2.859574468	
133	2021/1/8 8:46	Fri.	Day before a holiday	◯	80	Male	Neurosurg.	HH	WMUH	Transfer									
134	2021/1/14 14:47	Thu.			80	Male	EmergentCare M.	HMH	WMUH	No	55.4	110.8	1.108	2.466	45000	319.1489362	4653.6	33.00425532	
135	2021/1/15 9:32	Fri.	Day before a holiday		10	Male	EmergentCare M.	MWMC	WMUH	Transfer									
136	2021/1/15 12:20	Fri.	Day before a holiday		70	Male	EmergentCare M.	KGC	NMH	Transfer									
137	2021/1/22 3:38	Fri.	Day before a holiday	◯	70	Male	Cardiovasc.Surg.	HMH	WMUH	Transfer									
138	2021/1/22 11:56	Fri.	Day before a holiday		70	Female	Neurosurg.	KGC	HMH	Transfer									
139	2021/1/22 15:59	Fri.	Day before a holiday		90	Male	Cardiovasc.Surg.	HH	WMUH	No	41.3	82.6	0.826	1.902	45000	319.1489362	3469.2	24.60425532	
140	2021/1/26 19:48	Tue.		◯	70	Female	Neurosurg.	NTOH	SMMC	Transfer									
141	2021/1/27 14:33	Tue.			70	Female	EmergentCare M.	KGC	HMH	Transfer									
142	2021/2/2 12:45	Tue.			80	Male	Cardiovasc.Surg.	AMH	WMUH	Transfer									
143	2021/2/2 15:07	Tue.			70	Female	EmergentCare M.	KH	WMUH	Transfer									
144	2021/2/8 5:32	Mon.		◯	80	Male	Neurosurg.	NTOH	SMMC	Transfer									
145	2021/2/9 11:46	Tue.			80	Male	EmergentCare M.	HH	WMUH	Transfer									
146	2021/2/10 12:35	Wed.	Day before a holiday		70	Male	Cardiovasc.Surg.	RCWC	WMUH	Transfer									
147	2021/2/17 11:23	Wed.			60	Female	Neurosurg.	HH	WMUH	Transfer									
148	2021/2/18 11:24	Thu.			70	Female	Cardiovasc.Surg.	HH	WMUH	Transfer									
149	2021/2/24 23:08	Wed.		◯	30	Male	Neurosurg.	SMMC	WMUH	Transfer									
150	2021/2/25 3:55	Thu.		◯	70	Male	EmergentCare M.	RCWC	WMUH	Transfer									
151	2021/2/28 0:00	Sun.	Holiday	◯	70	Female	Neurosurg.	NTOH	SMMC	No	10.6	21.2	0.212	0.674	45000	319.1489362	890.4	6.314893617	
152	2021/3/2 15:17	Tue.			60	Male	Neurosurg.	HH	WMUH	No	41.3	82.6	0.826	1.902	45000	319.1489362	3469.2	24.60425532	
153	2021/3/5 11:17	Fri.	Day before a holiday		70	Male	EmergentCare M.	WMUH	RCWC	Transfer									
154	2021/3/5 11:37	Fri.	Day before a holiday		70	Female	EmergentCare M.	MWMC	WMUH	No	74.6	149.2	1.492	3.234	45000	319.1489362	6266.4	44.44255319	
155	2021/3/5 15:23	Fri.	Day before a holiday		80	Female	Neurosurg.	KGC	HMH	No	30.3	60.6	0.606	1.462	45000	319.1489362	2545.2	18.05106383	
156	2021/3/6 13:20	Sat.	Holiday		60	Male	Neurosurg.	KH	MWMC	Transfer									
157	2021/3/7 1:32	Sun.	Holiday	◯	60	Male	EmergentCare M.	RCWC	WMUH	No	4.7	9.4	0.094	0.438	45000	319.1489362	394.8	2.8	
158	2021/3/19 10:55	Fri.	Day before a holiday		70	Male	Neurosurg.	KMH	MWMC	Transfer									
159	2021/3/19 11:45	Fri.	Day before a holiday		80	Female	Neurosurg.	HH	WMUH	No	41.3	82.6	0.826	1.902	45000	319.1489362	3469.2	24.60425532	
160	2021/3/19 18:44	Fri.	Day before a holiday	◯	70	Male	Neurosurg.	KH	MWMC	No	4.8	9.6	0.096	0.442	45000	319.1489362	403.2	2.859574468	
161	2021/3/21 12:16	Sun.	Holiday		70	Female	Neurosurg.	HH	WMUH	Transfer									
162	2021/3/21 22:12	Sun.	Holiday	◯	60	Female	Neurosurg.	KH	MWMC	No	4.8	9.6	0.096	0.442	45000	319.1489362	403.2	2.859574468	
163	2021/3/21 23:16	Sun.	Holiday	◯	70	Male	EmergentCare M.	NTOH	MWMC	Transfer									
164	2021/3/24 14:34	Wed.			80	Female	EmergentCare M.	KGC	HMH	No	30.3	60.6	0.606	1.462	45000	319.1489362	2545.2	18.05106383	
165	2021/3/25 12:53	Thu.			70	Female	Cardiovasc.Surg.	RCWC	WMUH	Transfer									
166	2021/3/25 15:39	Thu.			80	Female	EmergentCare M.	KGC	HMH	No	30.3	60.6	0.606	1.462	45000	319.1489362	2545.2	18.05106383	
167	2021/3/26 22:06	Fri.	Day before a holiday	◯	50	Male	Neurosurg.	HH	WMUH	Transfer									
168	2021/3/29 13:17	Mon.			80	Female	EmergentCare M.	KH	RCWC	Transfer									
169	2021/3/29 16:06	Mon.			80	Female	EmergentCare M.	KGC	WMUH	Transfer									
170	2021/4/2 16:33	Fri.	Day before a holiday		60	Male	EmergentCare M.	HMH	WMUH	Transfer									
171	2021/4/5 10:20	Mon.			80	Female	Neurosurg.	KH	MWMC	Transfer									
172	2021/4/5 14:14	Mon.			80	Female	EmergentCare M.	NMH	WMUH	Transfer									
173	2021/4/7 16:19	Wed.			90	Male	Neurosurg.	KMH	MWMC	Transfer									
174	2021/4/12 13:07	Mon.			80	Female	EmergentCare M.	KGC	HMH	Transfer									
175	2021/4/12 15:45	Mon.			70	Male	Cardiovasc.Surg.	SMMC	KH	Transfer									
176	2021/4/15 13:36	Thu.			70	Male	Neurosurg.	KH	MWMC	No	4.8	9.6	0.096	0.442	45000	319.1489362	403.2	2.859574468	
177	2021/4/18 15:56	Sun.	Holiday		80	Male	EmergentCare M.	KH	MWMC	No	4.8	9.6	0.096	0.442	45000	319.1489362	403.2	2.859574468	
178	2021/4/21 14:03	Wed.			40	Female	EmergentCare M.	NTOH	MWMC	No	88.4	176.8	1.768	3.786	45000	319.1489362	7425.6	52.66382979	
179	2021/4/29 10:29	Thu.	Holiday		30	Male	Neurosurg.	KH	MWMC	No	4.8	9.6	0.096	0.442	45000	319.1489362	403.2	2.859574468	
180	2021/4/29 15:49	Thu.	Holiday		80	Male	Neurosurg.	KMH	SMMC	Transfer									
181	2021/4/29 17:29	Thu.	Holiday	◯	70	Male	Neurosurg.	KH	MWMC	Transfer									
182	2021/5/1 8:48	Sat.	Holiday	◯	100	Female	Neurosurg.	KH	MWMC	Transfer									
183	2021/5/3 17:29	Mon.	Holiday	◯	70	Female	EmergentCare M.	HH	WMUH	Transfer									
184	2021/5/7 10:03	Fri.	Day before a holiday		60	Male	EmergentCare M.	KGC	HMH	Transfer									
185	2021/5/13 12:22	Thu.			70	Male	Neurosurg.	KGC	HMH	Transfer									
186	2021/5/13 12:43	Thu.			80	Female	EmergentCare M.	KGC	WMUH	No	61.5	123	1.23	2.71	45000	319.1489362	5166	36.63829787	
187	2021/5/14 15:43	Fri.	Day before a holiday		60	Female	EmergentCare M.	RCWC	WMUH	Transfer									
188	2021/5/19 11:36	Wed.			80	Male	EmergentCare M.	KGC	HMH	Transfer									
189	2021/5/21 15:59	Fri.	Day before a holiday		60	Male	Neurosurg.	KMH	MWMC	Transfer									
190	2021/5/23 10:14	Sun.	Holiday		80	Female	EmergentCare M.	KGC	HMH	Transfer									
191	2021/5/23 13:41	Sun.	Holiday		50	Male	EmergentCare M.	KMH	SMMC	Transfer									
192	2021/5/23 15:20	Sun.	Holiday		80	Male	EmergentCare M.	HH	WMUH	Transfer									
193	2021/5/24 2:18	Mon.		◯	70	Male	EmergentCare M.	HH	RCWC	Transfer									
194	2021/5/25 9:27	Tue.			70	Male	Neurosurg.	KMH	MWMC	Transfer									
195	2021/5/25 13:53	Tue.			80	Female	EmergentCare M.	KGC	HMH	Transfer									
196	2021/5/30 21:06	Sun.	Holiday	◯	40	Female	Neurosurg.	KH	MWMC	No	4.8	9.6	0.096	0.442	45000	319.1489362	403.2	2.859574468	
197	2021/6/2 11:18	Wed.			80	Male	Cardiovasc.Surg.	NMH	WMUH	No	31.9	63.8	0.638	1.526	45000	319.1489362	2679.6	19.00425532	
198	2021/6/4 9:38	Fri.	Day before a holiday		30	Female	EmergentCare M.	RCWC	WMUH	Transfer									
199	2021/6/4 14:12	Fri.	Day before a holiday		60	Male	Neurosurg.	KH	MWMC	Transfer									
200	2021/6/7 17:40	Mon.		◯	60	Male	EmergentCare M.	KH	MWMC	Transfer									
201	2021/6/10 16:30	Thu.			60	Male	Cardiovasc.Surg.	HH	WMUH	Transfer									
202	2021/6/16 3:56	Wed.		◯	80	Male	EmergentCare M.	KMH	SMMC	Transfer									
203	2021/6/21 4:39	Mon.		◯	40	Male	EmergentCare M.	KH	WMUH	No	69.1	138.2	1.382	3.014	45000	319.1489362	5804.4	41.16595745	
204	2021/6/30 9:30	Wed.			70	Male	Neurosurg.	KH	MWMC	Transfer									
205	2021/7/4 19:55	Sun.	Holiday	◯	70	Male	EmergentCare M.	KGC	HMH	Transfer									
206	2021/7/6 15:06	Tue.			50	Female	Neurosurg.	KH	MWMC	Transfer									
207	2021/7/6 17:35	Tue.		◯	50	Female	Neurosurg.	MWMC	WMUH	Transfer									
208	2021/7/7 21:05	Wed.		◯	10	Male	Neurosurg.	MWMC	WMUH	Transfer									
209	2021/7/16 9:31	Fri.	Day before a holiday		10	Male	Cardiovasc.Surg.	KH	WMUH	Transfer									
210	2021/7/19 13:07	Mon.	Holiday		70	Male	EmergentCare M.	KGC	WMUH	Transfer									
211	2021/7/19 16:47	Mon.	Holiday		100	Female	Neurosurg.	KMH	SMMC	No	36.2	72.4	0.724	1.698	45000	319.1489362	3040.8	21.56595745	
212	2021/7/22 15:56	Thu.	Day before a holiday		60	Male	EmergentCare M.	HH	WMUH	Transfer									
213	2021/7/25 14:32	Sun.	Holiday		80	Female	EmergentCare M.	MWMC	WMUH	Transfer									
214	2021/7/26 12:55	Mon.			10	Male	EmergentCare M.	HH	WMUH	Transfer									
215	2021/7/28 15:02	Wed.			90	Female	Neurosurg.	KMH	SMMC	Transfer									
216	2021/7/29 11:58	Thu.			70	Female	EmergentCare M.	KGC	HMH	No	30.3	60.6	0.606	1.462	45000	319.1489362	2545.2	18.05106383	
217	2021/7/30 11:45	Fri.	Day before a holiday		40	Female	EmergentCare M.	KGC	HMH	Transfer									
218	2021/8/3 11:51	Tue.			80	Female	Neurosurg.	KMH	SMMC	Transfer									
219	2021/8/3 15:10	Tue.			80	Female	EmergentCare M.	KGC	HMH	Transfer									
220	2021/8/4 16:35	Wed.			70	Male	Neurosurg.	KMH	MWMC	Transfer									
221	2021/8/5 13:57	Thu.			80	Male	Neurosurg.	WMUH	RCWC	No	4.7	9.4	0.094	0.438	45000	319.1489362	394.8	2.8	
222	2021/8/6 15:04	Fri.	Day before a holiday		90	Female	Neurosurg.	KH	MWMC	No	4.8	9.6	0.096	0.442	45000	319.1489362	403.2	2.859574468	
223	2021/8/9 0:25	Mon.		◯	70	Male	EmergentCare M.	NTOH	SMMC	Transfer									
224	2021/8/11 3:56	Wed.	Holiday	◯	70	Male	EmergentCare M.	KMH	MWMC	Transfer									
225	2021/8/12 13:36	Thu.			30	Male	EmergentCare M.	KMH	MWMC	Transfer									
226	2021/8/18 20:21	Fri.	Day before a holiday	◯	90	Female	Neurosurg.	KMH	SMMC	No	36.2	72.4	0.724	1.698	45000	319.1489362	3040.8	21.56595745	
227	2021/8/24 17:10	Tue.		◯	80	Female	EmergentCare M.	KMH	SMMC	Transfer									
228	2021/8/26 11:47	Thu.			50	Female	Neurosurg.	KMH	WMUH	Transfer									
229	2021/8/29 10:16	Sun.	Holiday		80	Female	Neurosurg.	KMH	SMMC	Transfer									
230	2021/8/31 9:02	Tue.			60	Female	Neurosurg.	HH	RCWC	Transfer									
231	2021/8/31 19:32	Tue.		◯	70	Male	Neurosurg.	WMUH	RCWC	Transfer									
232	2021/9/1 16:27	Wed.			70	Male	Neurosurg.	HH	RCWC	Transfer									
233	2021/9/2 9:56	Thu.			80	Male	Neurosurg.	HH	WMUH	Transfer									
234	2021/9/3 16:03	Fri.	Day before a holiday		80	Female	CardioVasc.M.	HH	WMUH	No	41.3	82.6	0.826	1.902	45000	319.1489362	3469.2	24.60425532	
235	2021/9/9 12:32	Thu.			80	Female	Cardiovasc.Surg.	SH	KH	Transfer									
236	2021/9/13 12:11	Mon.			80	Female	Cardiovasc.Surg.	HH	WMUH	Transfer									
237	2021/9/17 11:27	Fri.	Day before a holiday		50	Male	EmergentCare M.	HH	WMUH	Transfer									
238	2021/9/17 16:08	Fri.	Day before a holiday		70	Male	EmergentCare M.	KGC	HMH	Transfer									
239	2021/9/25 21:27	Sat.	Holiday	◯	60	Male	Neurosurg.	HH	WMUH	Transfer									
240	2021/9/29 16:57	Wed.			90	Female	EmergentCare M.	KGC	HMH	Transfer									
241	2021/10/1 9:01	Fri.	Day before a holiday		40	Male	EmergentCare M.	KGC	HMH	Transfer									
242	2021/10/1 9:26	Fri.	Day before a holiday		90	Female	EmergentCare M.	KGC	HMH	Transfer									
243	2021/10/14 15:19	Thu.			10	Female	EmergentCare M.	SMMC	MWMC	No	94.2	188.4	1.884	4.018	45000	319.1489362	7912.8	56.11914894	
244	2021/10/14 16:41	Thu.			30	Male	Neurosurg.	KMH	MWMC	Transfer									
245	2021/10/16 10:43	Sat.	Holiday		80	Male	EmergentCare M.	KMH	SMMC	Transfer									
246	2021/10/16 14:20	Sat.	Holiday		70	Female	Neurosurg.	KH	MWMC	No	4.8	9.6	0.096	0.442	45000	319.1489362	403.2	2.859574468	
247	2021/10/22 16:35	Fri.	Day before a holiday		90	Male	Neurosurg.	KH	MWMC	Transfer									
248	2021/10/24 13:02	Sun.	Holiday		80	Male	Neurosurg.	KMH	SMMC	Transfer									
249	2021/10/25 4:37	Mon.		◯	70	Male	Neurosurg.	KMH	SMMC	Transfer									
250	2021/10/27 15:32	Wed.			20	Female	Neurosurg.	KH	MWMC	No	4.8	9.6	0.096	0.442	45000	319.1489362	403.2	2.859574468	
251	2021/10/28 11:44	Thu.			70	Male	EmergentCare M.	KGC	HMH	Transfer									
252	2021/10/29 4:44	Fri.	Day before a holiday	◯	40	Male	EmergentCare M.	KH	MWMC	Transfer									
253	2021/10/29 13:50	Fri.	Day before a holiday		40	Male	Neurosurg.	HH	WMUH	Transfer									
254	2021/10/29 14:27	Fri.	Day before a holiday		10	Male	EmergentCare M.	KGC	HMH	Transfer									
255	2021/10/29 15:11	Fri.	Day before a holiday		60	Male	EmergentCare M.	HH	WMUH	Transfer									
256	2021/10/29 19:43	Fri.	Day before a holiday	◯	70	Male	EmergentCare M.	WMUH	RCWC	No	4.7	9.4	0.094	0.438	45000	319.1489362	394.8	2.8	
257	2021/11/3 19:04	Wed.	Holiday	◯	70	Female	Cardiovasc.Surg.	NTOH	KH	Transfer									
258	2021/11/8 16:00	Mon.			10	Male	EmergentCare M.	KGC	HMH	No	30.3	60.6	0.606	1.462	45000	319.1489362	2545.2	18.05106383	
259	2021/11/8 21:42	Mon.		◯	70	Male	Neurosurg.	KH	MWMC	No	4.8	9.6	0.096	0.442	45000	319.1489362	403.2	2.859574468	
260	2021/11/9 20:32	Tue.		◯	70	Male	Neurosurg.	WMUH	RCWC	Transfer									
261	2021/11/13 22:41	Sat.	Holiday	◯	90	Female	EmergentCare M.	KMH	WMUH	Transfer									
262	2021/11/16 3:09	Tue.		◯	60	Male	EmergentCare M.	KMH	SMMC	Transfer									
263	2021/11/24 13:57	Wed.			70	Male	Cardiovasc.Surg.	NMH	WMUH	Transfer									
264	2021/11/29 17:48	Mon.		◯	50	Male	Neurosurg.	KH	MWMC	Transfer									
265	2021/12/3 17:09	Fri.	Day before a holiday	◯	70	Female	EmergentCare M.	HH	WMUH	Transfer									
266	2021/12/8 14:04	Wed.			60	Female	Neurosurg.	KGC	HMH	Transfer									
267	2021/12/13 9:33	Mon.			40	Male	EmergentCare M.	KMH	SMMC	Transfer									
268	2021/12/16 12:36	Thu.			60	Male	EmergentCare M.	KMH	WMUH	Transfer									
269	2021/12/19 3:30	Sun.	Holiday	◯	80	Female	Neurosurg.	NTOH	SMMC	No	10.6	21.2	0.212	0.674	45000	319.1489362	890.4	6.314893617	
270	2021/12/23 14:14	Thu.			80	Male	EmergentCare M.	KGC	HMH	Transfer									
271	2021/12/26 15:32	Sun.	Holiday		70	Female	Neurosurg.	WMUH	RCWC	Transfer									
272	2021/12/27 9:57	Mon.			80	Female	EmergentCare M.	KGC	HMH	Transfer									
273	2021/12/30 10:09	Thu.	Holiday		70	Male	EmergentCare M.	HH	WMUH	Transfer									
274	2021/12/30 20:09	Thu.	Holiday	◯	70	Female	Neurosurg.	KMH	SMMC	No	36.2	72.4	0.724	1.698	45000	319.1489362	3040.8	21.56595745	
275	2021/12/31 14:40	Fri.	Holiday		10	Male	Neurosurg.	HH	WMUH	No	41.3	82.6	0.826	1.902	45000	319.1489362	3469.2	24.60425532	
276	2022/1/3 12:06	Mon.	Holiday		50	Male	Cardiovasc.Surg.	HH	WMUH	Transfer									
277	2022/1/3 16:25	Mon.	Holiday		80	Female	EmergentCare M.	HH	WMUH	Transfer									
278	2022/1/4 13:22	Tue.			80	Female	EmergentCare M.	KGC	HMH	Transfer									
279	2022/1/6 8:29	Thu.		◯	60	Female	EmergentCare M.	KGC	HMH	Transfer									
280	2022/1/6 16:27	Thu.			90	Female	Neurosurg.	KH	MWMC	Transfer									
281	2022/1/6 17:46	Thu.		◯	70	Female	Neurosurg.	HH	WMUH	No	41.3	82.6	0.826	1.902	45000	319.1489362	3469.2	24.60425532	
282	2022/1/6 19:13	Thu.		◯	80	Female	Neurosurg.	NTOH	SMMC	Transfer									
283	2022/1/7 6:14	Fri.	Day before a holiday	◯	70	Male	EmergentCare M.	NTOH	SMMC	Transfer									
284	2022/1/13 11:26	Thu.			70	Male	EmergentCare M.	KGC	HMH	Transfer									
285	2022/1/17 13:51	Mon.			70	Male	Cardiovasc.Surg.	HH	WMUH	Transfer									
286	2022/1/19 10:15	Wed.			70	Male	EmergentCare M.	KGC	NMH	No	33.4	66.8	0.668	1.586	45000	319.1489362	2805.6	19.89787234	
287	2022/1/19 11:54	Wed.			60	Male	EmergentCare M.	NTOH	MWMC	No	88.4	176.8	1.768	3.786	45000	319.1489362	7425.6	52.66382979	
288	2022/1/21 10:05	Fri.	Day before a holiday		60	Male	EmergentCare M.	KGC	WMUH	Transfer									
289	2022/1/21 13:32	Fri.	Day before a holiday		60	Male	EmergentCare M.	NTOH	MWMC	No	88.4	176.8	1.768	3.786	45000	319.1489362	7425.6	52.66382979	
290	2022/1/24 11:00	Mon.			10	Male	Neurosurg.	KH	MWMC	Transfer									
291	2022/1/26 19:35	Wed.		◯	90	Female	Neurosurg.	KMH	SMMC	No	36.2	72.4	0.724	1.698	45000	319.1489362	3040.8	21.56595745	
292	2022/1/26 22:14	Wed.		◯	70	Male	Neurosurg.	KMH	SMMC	Transfer									
293	2022/1/30 8:04	Sun.	Holiday	◯	80	Female	Neurosurg.	NTOH	SMMC	Transfer									
294	2022/2/3 14:17	Thu.			80	Male	Neurosurg.	KMH	SMMC	Transfer									
295	2022/2/6 16:23	Sun.	Holiday		90	Female	EmergentCare M.	KGC	HMH	Transfer									
296	2022/2/7 10:29	Mon.			80	Male	EmergentCare M.	NTOH	SMMC	Transfer									
297	2022/2/8 16:14	Tue.			80	Male	EmergentCare M.	KGC	HMH	Transfer									
298	2022/2/9 13:58	Wed.			80	Male	Neurosurg.	HH	WMUH	Transfer									
299	2022/2/14 10:21	Mon.			40	Female	EmergentCare M.	KMH	SMMC	No	36.2	72.4	0.724	1.698	45000	319.1489362	3040.8	21.56595745	
300	2022/2/15 16:45	Tue.			70	Male	EmergentCare M.	NTOH	SMMC	No	10.6	21.2	0.212	0.674	45000	319.1489362	890.4	6.314893617	
301	2022/2/16 1:35	Wed.		◯	50	Male	Cardiovasc.Surg.	NTOH	SMMC	Transfer									
302	2022/2/16 15:23	Wed.			60	Male	Neurosurg.	NTOH	SMMC	No	10.6	21.2	0.212	0.674	45000	319.1489362	890.4	6.314893617	
303	2022/2/17 6:09	Thu.		◯	10	Female	EmergentCare M.	KMH	SMMC	Transfer									
304	2022/2/19 19:59	Sat.	Holiday	◯	80	Male	EmergentCare M.	HH	WMUH	Transfer									
305	2022/2/21 11:05	Mon.			60	Female	EmergentCare M.	KGC	HMH	Transfer									
306	2022/2/24 13:32	Thu.			70	Male	EmergentCare M.	HH	WMUH	Transfer									
307	2022/2/25 8:40	Fri.	Day before a holiday	◯	80	Male	Neurosurg.	KH	MWMC	No	4.8	9.6	0.096	0.442	45000	319.1489362	403.2	2.859574468	
308	2022/2/27 9:50	Sun.	Holiday		40	Male	Neurosurg.	SMMC	WMUH	Transfer									
309	2022/3/2 9:11	Wed.			80	Male	Cardiovasc.Surg.	NTOH	SMMC	No	10.6	21.2	0.212	0.674	45000	319.1489362	890.4	6.314893617	
310	2022/3/2 9:59	Wed.			80	Male	EmergentCare M.	SMMC	MWMC	Transfer									
311	2022/3/2 13:12	Wed.			80	Female	Cardiovasc.Surg.	HH	WMUH	Transfer									
312	2022/3/2 16:09	Wed.			80	Female	EmergentCare M.	KGC	HMH	Transfer									
313	2022/3/4 16:24	Fri.	Day before a holiday		80	Male	Neurosurg.	KH	MWMC	Transfer									
314	2022/3/11 2:51	Fri.	Day before a holiday	◯	80	Female	Neurosurg.	KMH	SMMC	No	36.2	72.4	0.724	1.698	45000	319.1489362	3040.8	21.56595745	
315	2022/3/11 17:10	Fri.	Day before a holiday	◯	80	Female	Neurosurg.	KMH	SMMC	No	36.2	72.4	0.724	1.698	45000	319.1489362	3040.8	21.56595745	
316	2022/3/13 9:55	Sun.	Holiday		80	Female	EmergentCare M.	HH	WMUH	Transfer									
317	2022/3/14 23:30	Mon.		◯	70	Male	Neurosurg.	KH	MWMC	No	4.8	9.6	0.096	0.442	45000	319.1489362	403.2	2.859574468	
318	2022/3/17 14:54	Thu.			40	Female	Neurosurg.	KMH	MWMC	Transfer									
319	2022/3/20 13:08	Sun.	Holiday		80	Male	EmergentCare M.	NTOH	SMMC	No	10.6	21.2	0.212	0.674	45000	319.1489362	890.4	6.314893617	
320	2022/3/25 12:01	Fri.	Day before a holiday		90	Female	Neurosurg.	NTOH	SMMC	Transfer									
321	2022/3/25 18:00	Fri.	Day before a holiday	◯	80	Male	EmergentCare M.	KGC	HMH	Transfer									
322	2022/3/27 12:10	Sun.	Holiday		70	Male	Neurosurg.	HH	WMUH	Transfer									
323	2022/3/28 15:16	Mon.			80	Male	Neurosurg.	KMH	MWMC	Transfer									
324	2022/3/29 10:01	Tue.			70	Female	Cardiovasc.Surg.	HH	WMUH	No	41.3	82.6	0.826	1.902	45000	319.1489362	3469.2	24.60425532	
325	2022/3/30 12:00	Wed.			80	Male	Neurosurg.	KH	MWMC	Transfer									
326	2022/4/5 15:04	Tue.			80	Male	CardioVasc.M.	KGC	HMH	Transfer	50	100	1	2					
327	2022/4/11 14:46	Mon.			60	Male	Neurosurg.	KGC	WMUH	Transfer		0							
328	2022/4/14 14:48	Thu.			50	Female	Cardiovasc.Surg.	NMH	WMUH	Transfer		0							
329	2022/4/18 12:16	Mon.			70	Female	Neurosurg.	NTOH	SMMC	Transfer		0							
330	2022/4/21 9:08	Thu.			70	Female	EmergentCare M.	NTOH	SMMC	Transfer		0							
331	2022/4/22 14:15	Fri.	Day before a holiday		80	Male	EmergentCare M.	KGC	HMH	Transfer		0							
332	2022/4/25 10:41	Mon.			10	Male	EmergentCare M.	KGC	HMH	Transfer		0							
333	2022/5/3 13:18	Tue.	Holiday		50	Male	Neurosurg.	KH	MWMC	Transfer		0							
334	2022/5/4 19:48	Wed.	Holiday	◯	80	Male	Neurosurg.	KMH	SMMC	No	36.2	72.4	0.724	1.698	45000	319.1489362	3040.8	21.56595745	
335	2022/5/6 21:57	Fri.	Day before a holiday	◯	90	Female	Cardiovasc.Surg.	KMH	SMMC	Transfer		0							
336	2022/5/9 15:24	Mon.			90	Male	EmergentCare M.	KMH	SMMC	Transfer		0							
337	2022/5/13 14:04	Fri.	Day before a holiday		40	Male	Neurosurg.	KGC	HMH	Transfer		0							
338	2022/5/18 14:32	Wed.			50	Female	Neurosurg.	NTOH	SMMC	Transfer		0							
339	2022/5/20 13:56	Fri.	Day before a holiday		70	Female	Neurosurg.	KGC	WMUH	Transfer		0							
340	2022/5/20 13:30	Fri.	Day before a holiday		70	Female	Cardiovasc.Surg.	RCWC	WMUH	Transfer		0							
341	2022/5/21 16:48	Sat.	Holiday		60	Male	Neurosurg.	NTOH	SMMC	Transfer		0							
342	2022/5/24 14:26	Tue.			80	Female	EmergentCare M.	HH	WMUH	Transfer		0							
343	2022/5/30 9:23	Mon.			70	Male	Neurosurg.	NTOH	SMMC	No	10.6	21.2	0.212	0.674	45000	319.1489362	890.4	6.314893617	
344	2022/5/30 18:00	Mon.		◯	80	Male	EmergentCare M.	KGC	NMH	Transfer		0							
345	2022/5/31 12:36	Tue.			60	Male	Neurosurg.	NTOH	SMMC	Transfer		0							
346	2022/6/2 15:54	Thu.			70	Male	Neurosurg.	NTOH	SMMC	No	10.6	21.2	0.212	0.674	45000	319.1489362	890.4	6.314893617	
347	2022/6/5 8:35	Sun.	Holiday	◯	60	Male	Neurosurg.	KH	MWMC	Transfer		0							
348	2022/6/6 1:06	Mon.		◯	70	Male	EmergentCare M.	RCWC	WMUH	Transfer		0							
349	2022/6/6 12:02	Mon.			20	Male	EmergentCare M.	KGC	HMH	Transfer		0							
350	2022/6/12 17:25	Sun.	Holiday	◯	50	Female	Neurosurg.	KH	WMUH	No	69.1	138.2	1.382	3.014	45000	319.1489362	5804.4	41.16595745	
351	2022/6/13 11:32	Mon.			60	Female	Cardiovasc.Surg.	KGC	HMH	Transfer		0							
352	2022/6/13 17:41	Mon.		◯	70	Male	EmergentCare M.	KGC	HMH	Transfer		0							
353	2022/6/17 12:10	Fri.	Day before a holiday		80	Male	Neurosurg.	NTOH	SMMC	No	10.6	21.2	0.212	0.674	45000	319.1489362	890.4	6.314893617	
354	2022/6/19 15:16	Sun.	Holiday		70	Male	Neurosurg.	KMH	SMMC	No	36.2	72.4	0.724	1.698	45000	319.1489362	3040.8	21.56595745	
355	2022/6/20 15:20	Mon.			80	Male	Neurosurg.	KMH	SMMC	Transfer		0							
356	2022/6/28 11:24	Tue.			90	Female	EmergentCare M.	KGC	HMH	Transfer		0							
357	2022/6/30 7:45	Thu.		◯	70	Male	Neurosurg.	NTOH	SMMC	No	10.6	21.2	0.212	0.674	45000	319.1489362	890.4	6.314893617	
358	2022/6/30 19:43	Thu.		◯	80	Female	Neurosurg.	NTOH	SMMC	No	10.6	21.2	0.212	0.674	45000	319.1489362	890.4	6.314893617	
359	2022/7/5 10:26	Tue.			80	Female	Neurosurg.	NTOH	SMMC	No	10.6	21.2	0.212	0.674	45000	319.1489362	890.4	6.314893617	
360	2022/7/6 14:36	Wed.			50	Female	EmergentCare M.	KGC	HMH	Transfer		0							
361	2022/7/7 16:53	Thu.			80	Female	Neurosurg.	KGC	HMH	Transfer		0							
362	2022/7/8 2:22	Fri.	Day before a holiday	◯	60	Male	EmergentCare M.	NMH	WMUH	Transfer		0							
363	2022/7/9 10:42	Sat.	Holiday		50	Male	EmergentCare M.	NMH	WMUH	Transfer		0							
364	2022/7/11 10:00	Mon.			70	Female	EmergentCare M.	NTOH	SMMC	Transfer		0							
365	2022/7/11 11:44	Mon.			80	Male	Cardiovasc.Surg.	WMUH	RCWC	No	4.7	9.4	0.094	0.438	45000	319.1489362	394.8	2.8	
366	2022/7/15 15:17	Fri.	Day before a holiday		80	Female	EmergentCare M.	NMH	WMUH	Transfer		0							
367	2022/7/16 20:07	Sat.	Holiday	◯	50	Female	EmergentCare M.	RCWC	WMUH	Transfer		0							
368	2022/7/20 15:50	Wed.			60	Female	Neurosurg.	NTOH	SMMC	Transfer		0							
369	2022/7/21 16:46	Thu.			50	Female	EmergentCare M.	NTOH	SMMC	Transfer		0							
370	2022/7/22 12:35	Fri.	Day before a holiday		80	Female	Neurosurg.	NTOH	SMMC	No	10.6	21.2	0.212	0.674	45000	319.1489362	890.4	6.314893617	
371	2022/7/25 8:57	Mon.		◯	80	Male	Neurosurg.	KMH	SMMC	Transfer		0							
372	2022/7/25 14:30	Mon.			70	Female	EmergentCare M.	NTOH	SMMC	Transfer		0							
373	2022/7/27 11:20	Wed.			70	Male	Neurosurg.	NTOH	SMMC	No	10.6	21.2	0.212	0.674	45000	319.1489362	890.4	6.314893617	
374	2022/7/28 10:10	Thu.			70	Male	Neurosurg.	NTOH	SMMC	No	10.6	21.2	0.212	0.674	45000	319.1489362	890.4	6.314893617	
375	2022/8/3 16:42	Wed.			80	Female	Cardiovasc.Surg.	NTOH	SMMC	Transfer		0							
376	2022/8/4 14:42	Thu.			50	Male	EmergentCare M.	HH	WMUH	Transfer		0							
377	2022/8/8 13:16	Mon.			70	Female	Neurosurg.	NTOH	SMMC	No	10.6	21.2	0.212	0.674	45000	319.1489362	890.4	6.314893617	
378	2022/8/9 22:46	Tue.		◯	40	Female	EmergentCare M.	NTOH	SMMC	Transfer		0							
379	2022/8/10 12:55	Wed.	Day before a holiday		90	Male	Cardiovasc.Surg.	NTOH	SMMC	Transfer		0							
380	2022/8/14 14:30	Sun.	Holiday		10	Male	EmergentCare M.	RCWC	WMUH	Transfer		0							
381	2022/8/15 11:18	Mon.			90	Female	EmergentCare M.	NTOH	SMMC	No	10.6	21.2	0.212	0.674	45000	319.1489362	890.4	6.314893617	
382	2022/8/18 11:48	Thu.			80	Male	EmergentCare M.	KGC	NMH	Transfer		0							
383	2022/8/19 18:46	Fri.	Day before a holiday	◯	70	Male	EmergentCare M.	NTOH	SMMC	Transfer		0							
384	2022/8/22 9:01	Mon.			50	Male	Neurosurg.	KMH	SMMC	Transfer		0							
385	2022/8/22 10:28	Mon.			70	Female	EmergentCare M.	NTOH	SMMC	Transfer		0							
386	2022/8/24 9:40	Wed.			40	Female	Neurosurg.	KMH	MWMC	Transfer		0							
387	2022/8/26 8:15	Fri.	Day before a holiday	◯	50	Female	Neurosurg.	NTOH	SMMC	No	10.6	21.2	0.212	0.674	45000	319.1489362	890.4	6.314893617	
388	2022/8/28 13:10	Sun.	Holiday		90	Female	Neurosurg.	NTOH	SMMC	No	10.6	21.2	0.212	0.674	45000	319.1489362	890.4	6.314893617	
389	2022/8/29 14:39	Mon.			80	Female	EmergentCare M.	NTOH	SMMC	No	10.6	21.2	0.212	0.674	45000	319.1489362	890.4	6.314893617	
390	2022/9/1 15:12	Thu.			90	Male	Neurosurg.	NTOH	SMMC	No	10.6	21.2	0.212	0.674	45000	319.1489362	890.4	6.314893617	
391	2022/9/2 20:14	Fri.	Day before a holiday	◯	40	Female	EmergentCare M.	HH	WMUH	Transfer		0							
392	2022/9/6 14:36	Tue.			70	Male	EmergentCare M.	NTOH	SMMC	No	10.6	21.2	0.212	0.674	45000	319.1489362	890.4	6.314893617	
393	2022/9/7 9:50	Wed.			70	Male	EmergentCare M.	NTOH	SMMC	Transfer		0							
394	2022/9/9 20:17	Fri.	Day before a holiday	◯	60	Male	Neurosurg.	NMH	WMUH	Transfer		0							
395	2022/9/11 14:46	Sun.	Holiday		70	Female	EmergentCare M.	KGC	HMH	Transfer		0							
396	2022/9/15 12:52	Thu.			80	Female	Neurosurg.	KMH	MWMC	No	60.3	120.6	1.206	2.662	45000	319.1489362	5065.2	35.92340426	
397	2022/9/21 9:04	Wed.			80	Male	EmergentCare M.	NTOH	SMMC	Transfer		0							
398	2022/9/22 12:48	Thu.	Day before a holiday		70	Male	EmergentCare M.	KGC	HMH	Transfer		0							
399	2022/9/23 11:12	Fri.	Holiday		90	Male	EmergentCare M.	NTOH	SMMC	No	10.6	21.2	0.212	0.674	45000	319.1489362	890.4	6.314893617	
400	2022/9/30 15:53	Fri.	Day before a holiday		70	Male	Neurosurg.	KH	MWMC	Transfer		0							
401	2022/10/1 15:37	Sat.	Holiday		90	Female	Neurosurg.	KH	WMUH	No	69.1	138.2	1.382	3.014	45000	319.1489362	5804.4	41.16595745	
402	2022/10/3 16:41	Mon.			80	Male	EmergentCare M.	KGC	HMH	Transfer		0							
403	2022/10/4 15:15	Tue.			80	Male	EmergentCare M.	NTOH	MWMC	No	88.4	176.8	1.768	3.786	45000	319.1489362	7425.6	52.66382979	
404	2022/10/5 10:27	Wed.			80	Male	EmergentCare M.	NTOH	SMMC	No	10.6	21.2	0.212	0.674	45000	319.1489362	890.4	6.314893617	
405	2022/10/5 15:16	Wed.			60	Male	Neurosurg.	NTOH	SMMC	No	10.6	21.2	0.212	0.674	45000	319.1489362	890.4	6.314893617	
406	2022/10/7 16:12	Fri.	Day before a holiday		20	Male	EmergentCare M.	KMH	MWMC	Transfer		0							
407	2022/10/7 15:23	Fri.	Day before a holiday		70	Male	Cardiovasc.Surg.	WMUH	RCWC	No	4.7	9.4	0.094	0.438	45000	319.1489362	394.8	2.8	
408	2022/10/8 23:50	Sat.	Holiday	◯	80	Female	EmergentCare M.	KMH	SMMC	Transfer		0							
409	2022/10/9 13:14	Sun.	Holiday		40	Female	EmergentCare M.	NTOH	SMMC	Transfer		0							
410	2022/10/11 9:09	Tue.			80	Female	Neurosurg.	NTOH	SMMC	No	10.6	21.2	0.212	0.674	45000	319.1489362	890.4	6.314893617	
411	2022/10/11 11:45	Tue.			80	Female	Neurosurg.	NTOH	SMMC	No	10.6	21.2	0.212	0.674	45000	319.1489362	890.4	6.314893617	
412	2022/10/12 11:24	Wed.			70	Male	Neurosurg.	NTOH	SMMC	No	10.6	21.2	0.212	0.674	45000	319.1489362	890.4	6.314893617	
413	2022/10/13 16:32	Thu.			70	Female	EmergentCare M.	NTOH	SMMC	Transfer		0							
414	2022/10/17 12:41	Mon.			80	Female	Neurosurg.	NTOH	SMMC	No	10.6	21.2	0.212	0.674	45000	319.1489362	890.4	6.314893617	
415	2022/10/18 13:35	Tue.			80	Female	Neurosurg.	NTOH	SMMC	No	10.6	21.2	0.212	0.674	45000	319.1489362	890.4	6.314893617	
416	2022/10/19 10:44	Wed.			70	Male	EmergentCare M.	NTOH	SMMC	Transfer		0							
417	2022/10/22 10:43	Sat.	Holiday		80	Male	Neurosurg.	KH	MWMC	Transfer		0							
418	2022/10/24 15:12	Mon.			70	Male	EmergentCare M.	NTOH	WMUH	No	158	316	3.16	6.57	45000	319.1489362	13272	94.12765957	
419	2022/10/25 10:49	Tue.			10	Male	EmergentCare M.	KGC	HMH	Transfer		0							
420	2022/10/25 15:09	Tue.			80	Male	EmergentCare M.	HH	WMUH	Transfer		0							
421	2022/10/26 21:10	Wed.		◯	60	Female	Neurosurg.	HH	WMUH	Transfer		0							
422	2022/10/27 15:01	Thu.			80	Male	EmergentCare M.	NTOH	SMMC	Transfer		0							
423	2022/10/29 0:20	Sat.	Holiday	◯	80	Female	EmergentCare M.	KMH	SMMC	Transfer		0							
424	2022/10/29 23:12	Sat.	Holiday	◯	70	Male	Neurosurg.	KMH	MWMC	Transfer		0							
425	2022/10/30 12:24	Sun.	Holiday		10	Female	EmergentCare M.	NTOH	SMMC	No	10.6	21.2	0.212	0.674	45000	319.1489362	890.4	6.314893617	
426	2022/11/4 13:30	Fri.	Day before a holiday		10	Male	EmergentCare M.	KMH	MWMC	Transfer		0							
427	2022/11/4 13:39	Fri.	Day before a holiday		50	Female	Neurosurg.	NTOH	SMMC	No	10.6	21.2	0.212	0.674	45000	319.1489362	890.4	6.314893617	
428	2022/11/5 13:58	Sat.	Holiday		70	Male	Neurosurg.	KH	MWMC	No	4.8	9.6	0.096	0.442	45000	319.1489362	403.2	2.859574468	
429	2022/11/6 6:08	Sun.	Holiday	◯	70	Male	Neurosurg.	KH	MWMC	Transfer		0							
430	2022/11/8 9:56	Tue.			80	Female	Neurosurg.	NTOH	SMMC	No	10.6	21.2	0.212	0.674	45000	319.1489362	890.4	6.314893617	
431	2022/11/10 12:58	Thu.			60	Male	Neurosurg.	NTOH	SMMC	No	10.6	21.2	0.212	0.674	45000	319.1489362	890.4	6.314893617	
432	2022/11/11 10:53	Fri.	Day before a holiday		70	Male	EmergentCare M.	NTOH	WMUH	Transfer		0							
433	2022/11/13 11:47	Sun.	Holiday		90	Female	Neurosurg.	NTOH	SMMC	No	10.6	21.2	0.212	0.674	45000	319.1489362	890.4	6.314893617	
434	2022/11/16 11:47	Wed.			90	Female	Neurosurg.	NTOH	SMMC	Transfer		0							
435	2022/11/17 9:06	Thu.			70	Female	EmergentCare M.	NTOH	SMMC	Transfer		0							
436	2022/11/17 16:33	Thu.			60	Female	EmergentCare M.	KGC	HMH	Transfer		0							
437	2022/11/18 11:58	Fri.	Day before a holiday		60	Male	Neurosurg.	NTOH	SMMC	No	10.6	21.2	0.212	0.674	45000	319.1489362	890.4	6.314893617	
438	2022/11/18 13:32	Fri.	Day before a holiday		50	Female	Neurosurg.	NTOH	SMMC	No	10.6	21.2	0.212	0.674	45000	319.1489362	890.4	6.314893617	
439	2022/11/19 22:05	Sat.	Holiday	◯	70	Male	Cardiovasc.Surg.	HH	WMUH	Transfer		0							
440	2022/11/21 11:30	Mon.			80	Male	EmergentCare M.	KGC	HMH	Transfer		0							
441	2022/11/24 14:58	Thu.			70	Male	Neurosurg.	KMH	MWMC	Transfer		0							
442	2022/11/24 14:38	Thu.			40	Male	EmergentCare M.	NTOH	SMMC	Transfer		0							
443	2022/11/26 11:58	Sat.	Holiday		80	Male	EmergentCare M.	KMH	SMMC	Transfer		0							
444	2022/11/28 18:10	Mon.		◯	80	Male	Neurosurg.	NTOH	SMMC	No	10.6	21.2	0.212	0.674	45000	319.1489362	890.4	6.314893617	
445	2022/11/29 10:36	Tue.			70	Male	Neurosurg.	NTOH	SMMC	No	10.6	21.2	0.212	0.674	45000	319.1489362	890.4	6.314893617	
446	2022/11/29 14:03	Tue.			80	Male	EmergentCare M.	NTOH	SMMC	Transfer		0							
447	2022/12/5 11:06	Mon.			70	Female	EmergentCare M.	NTOH	SMMC	No	10.6	21.2	0.212	0.674	45000	319.1489362	890.4	6.314893617	
448	2022/12/5 13:43	Mon.			80	Male	Neurosurg.	KH	MWMC	Transfer		0							
449	2022/12/6 14:48	Tue.			80	Female	Neurosurg.	KMH	SMMC	Transfer		0							
450	2022/12/7 18:03	Wed.		◯	90	Male	Neurosurg.	KH	MWMC	Transfer		0							
451	2022/12/7 18:48	Wed.		◯	80	Male	Neurosurg.	WMUH	RCWC	Transfer		0							
452	2022/12/9 8:52	Fri.	Day before a holiday	◯	90	Male	Neurosurg.	KMH	SMMC	Transfer		0							
453	2022/12/11 12:09	Sun.	Holiday		80	Male	EmergentCare M.	NTOH	SMMC	No	10.6	21.2	0.212	0.674	45000	319.1489362	890.4	6.314893617	
454	2022/12/11 21:09	Sun.	Holiday	◯	80	Female	Neurosurg.	NTOH	SMMC	No	10.6	21.2	0.212	0.674	45000	319.1489362	890.4	6.314893617	
455	2022/12/12 16:05	Mon.			60	Male	Neurosurg.	KGC	HMH	Transfer		0							
456	2022/12/12 17:00	Mon.		◯	80	Female	Neurosurg.	KH	MWMC	Transfer		0							
457	2022/12/14 7:44	Wed.		◯	50	Male	Cardiovasc.Surg.	NTOH	SMMC	Transfer		0							
458	2022/12/15 23:00	Thu.		◯	80	Male	EmergentCare M.	WMUH	RCWC	Transfer		0							
459	2022/12/16 11:23	Fri.	Day before a holiday		60	Male	EmergentCare M.	KGC	HMH	No	30.3	60.6	0.606	1.462	45000	319.1489362	2545.2	18.05106383	
460	2022/12/17 13:30	Sat.	Holiday		90	Female	CardioVasc.M.	NTOH	SMMC	Transfer		0							
461	2022/12/20 19:00	Tue.		◯	80	Female	EmergentCare M.	WMUH	RCWC	Transfer		0							
462	2022/12/21 18:01	Wed.		◯	50	Female	EmergentCare M.	NTOH	MWMC	Transfer		0							
463	2022/12/23 9:14	Fri.	Day before a holiday		70	Male	Neurosurg.	KGC	HMH	Transfer		0							
464	2022/12/23 8:20	Fri.	Day before a holiday	◯	70	Male	Neurosurg.	KMH	MWMC	Transfer		0							
465	2022/12/26 11:45	Mon.			80	Male	Neurosurg.	KGC	HMH	Transfer		0							
466	2022/12/26 7:35	Mon.		◯	70	Female	Neurosurg.	NTOH	SMMC	No	10.6	21.2	0.212	0.674	45000	319.1489362	890.4	6.314893617	
467	2022/12/26 16:09	Mon.			80	Male	EmergentCare M.	KGC	HMH	Transfer		0							
468	2022/12/28 13:45	Wed.	Day before a holiday		70	Female	Neurosurg.	KGC	HMH	Transfer		0							
469	2023/1/3 20:16	Tue.	Holiday	◯	80	Male	EmergentCare M.	NTOH	SMMC	Transfer		0							
470	2023/1/3 12:06	Wed.			80	Male	EmergentCare M.	KH	MWMC	No	4.8	9.6	0.096	0.442	45000	319.1489362	403.2	2.859574468	
471	2023/1/5 17:04	Thu.		◯	70	Male	Neurosurg.	NTOH	SMMC	No	10.6	21.2	0.212	0.674	45000	319.1489362	890.4	6.314893617	
472	2023/1/6 15:17	Fri.	Day before a holiday		80	Female	EmergentCare M.	NTOH	SMMC	No	10.6	21.2	0.212	0.674	45000	319.1489362	890.4	6.314893617	
473	2023/1/7 15:06	Sat.	Holiday		70	Male	Neurosurg.	KH	MWMC	Transfer		0							
474	2023/1/9 17:55	Mon.	Holiday	◯	80	Female	EmergentCare M.	KGC	HMH	Transfer		0							
475	2023/1/10 16:02	Tue.			70	Male	Neurosurg.	KMH	MWMC	Transfer		0							
476	2023/1/13 13:31	Fri.	Day before a holiday		70	Male	Neurosurg.	NTOH	SMMC	No	10.6	21.2	0.212	0.674	45000	319.1489362	890.4	6.314893617	
477	2023/1/20 13:12	Fri.	Day before a holiday		90	Male	EmergentCare M.	NTOH	SMMC	Transfer		0							
478	2023/1/23 13:17	Mon.			60	Male	Neurosurg.	NTOH	SMMC	No	10.6	21.2	0.212	0.674	45000	319.1489362	890.4	6.314893617	
479	2023/1/23 16:10	Mon.			80	Female	Neurosurg.	NTOH	SMMC	No	10.6	21.2	0.212	0.674	45000	319.1489362	890.4	6.314893617	
480	2023/1/24 13:54	Tue.			90	Female	Neurosurg.	NTOH	SMMC	No	10.6	21.2	0.212	0.674	45000	319.1489362	890.4	6.314893617	
481	2023/1/25 14:09	Wed.			60	Male	Neurosurg.	KMH	MWMC	Transfer		0							
482	2023/1/26 19:54	Thu.		◯	80	Male	Neurosurg.	HH	WMUH	No	41.3	82.6	0.826	1.902	45000	319.1489362	3469.2	24.60425532	
483	2023/1/28 19:09	Sat.	Holiday	◯	70	Male	Cardiovasc.Surg.	HH	WMUH	Transfer		0							
484	2023/1/31 9:33	Tue.			80	Male	EmergentCare M.	NTOH	SMMC	Transfer		0							
485	2023/2/2 14:43	Thu.			40	Male	EmergentCare M.	NTOH	MWMC	Transfer		0							
486	2023/2/6 16:00	Mon.			60	Male	EmergentCare M.	KMH	MWMC	Transfer		0							
487	2023/2/6 23:40	Mon.		◯	50	Male	Neurosurg.	KH	MWMC	Transfer		0							
488	2023/2/7 13:44	Tue.			90	Female	EmergentCare M.	NTOH	SMMC	No	10.6	21.2	0.212	0.674	45000	319.1489362	890.4	6.314893617	
489	2023/2/8 11:16	Wed.			70	Male	Neurosurg.	NTOH	SMMC	Transfer		0							
490	2023/2/8 12:51	Wed.			70	Male	Neurosurg.	NTOH	SMMC	No	10.6	21.2	0.212	0.674	45000	319.1489362	890.4	6.314893617	
491	2023/2/10 12:17	Fri.	Day before a holiday		80	Female	EmergentCare M.	NTOH	SMMC	No	10.6	21.2	0.212	0.674	45000	319.1489362	890.4	6.314893617	
492	2023/2/11 17:46	Sat.	Holiday	◯	90	Female	Neurosurg.	NTOH	SMMC	No	10.6	21.2	0.212	0.674	45000	319.1489362	890.4	6.314893617	
493	2023/2/13 20:41	Mon.		◯	90	Female	EmergentCare M.	NTOH	SMMC	Transfer		0							
494	2023/2/17 11:07	Fri.	Day before a holiday		70	Female	EmergentCare M.	NTOH	SMMC	No	10.6	21.2	0.212	0.674	45000	319.1489362	890.4	6.314893617	
495	2023/2/18 18:53	Sat.	Holiday	◯	80	Male	EmergentCare M.	KMH	SMMC	Transfer		0							
496	2023/2/21 12:19	Tue.			40	Male	Neurosurg.	NMH	WMUH	Transfer		0							
497	2023/2/25 12:25	Sat.	Holiday		90	Female	Neurosurg.	NTOH	SMMC	No	10.6	21.2	0.212	0.674	45000	319.1489362	890.4	6.314893617	
											3787.2	7574.4	75.744	188.488	6660000	47234.04255	313924.8	2226.417021	Total
